# Identification of FtfL as a novel target of berberine in intestinal bacteria

**DOI:** 10.1186/s12915-023-01778-w

**Published:** 2023-12-05

**Authors:** Jinci Yan, Chengli Fang, Gaohua Yang, Jianxu Li, Yanqiang Liu, Lu Zhang, Pengjie Yang, Jingyuan Fang, Yang Gu, Yu Zhang, Weihong Jiang

**Affiliations:** 1 CAS-Key Laboratory of Synthetic Biology, CAS Center for Excellence in Molecular Plant Sciences, Shanghai Institute of Plant Physiology and Ecology, Chinese Academy of Sciences, Shanghai, 200032 China; 2 University of Chinese Academy of Sciences, Beijing, China; 3 The Wallenberg Laboratory, Department of Molecular and Clinical Medicine, Sahlgrenska Academy, University of Gothenburg, Bruna Straket 16, 41345 Gothenburg, Sweden; 4 Division of Gastroenterology and Hepatology Key Laboratory of Gastroenterology & Hepatology, State Key Laboratory for Oncogenes and Related GenesSchool of Medicine, Ministry of Health, Renji Hospital, Shanghai Jiao-Tong University; Shanghai Institute of Digestive Disease, 145 Middle Shandong Road, Shanghai, 200001 China

**Keywords:** BBR, CRC, Intestinal microbes, Molecular target, Formate tetrahydrofolate ligase

## Abstract

**Background:**

Berberine (BBR) is a commonly used anti-intestinal inflammation drug, and its anti-cancer activity has been found recently. BBR can intervene and control malignant colorectal cancer (CRC) through intestinal microbes, but the direct molecular target and related mechanism are unclear. This study aimed to identify the target of BBR and dissect related mechanisms against the occurrence and development of CRC from the perspective of intestinal microorganisms.

**Results:**

Here, we found that BBR inhibits the growth of several CRC-driving bacteria, especially *Peptostreptococcus anaerobius*. By using a biotin-conjugated BBR derivative, we identified the protein FtfL (formate tetrahydrofolate ligase), a key enzyme in C1 metabolism, is the molecular target of BBR in *P. anaerobius*. BBR exhibits strong binding affinity and potent inhibition on FtfL. Based on this, we determined the crystal structure of *Pa*FtfL (*P. anaerobius* FtfL)-BBR complex and found that BBR can not only interfere with the conformational flexibility of *Pa*FtfL tetramer by wedging the tetramer interface but also compete with its substrate ATP for binding within the active center. In addition, the enzymatic activities of FtfL homologous proteins in human tumor cells can also be inhibited by BBR.

**Conclusions:**

In summary, our study has identified FtfL as a direct target of BBR and uncovered molecular mechanisms involved in the anti-CRC of BBR. BBR interferes with intestinal pathogenic bacteria by targeting FtfLs, suggesting a new means for controlling the occurrence and development of CRC.

**Supplementary Information:**

The online version contains supplementary material available at 10.1186/s12915-023-01778-w.

## Background

Colorectal cancer (CRC) is one of the malignant tumors with high incidence and mortality worldwide, accounting for about 10% of all cancers, ranking third in incidence [1]. The causes and pathogenesis of this disease are complex and diverse, which are closely related to intestinal microbes [2, 3]. Large-scale metagenomic sequencing analyses showed that the intestinal microbial composition of CRC patients is different from that of healthy individuals. Among them, *Peptostreptococcus anaerobius*, *Fusobacterium nucleatum*, and enterotoxigenic *Bacteroides fragilis* have obviously the highest abundances [4–6]. These microorganisms can cause the occurrence and development of CRC in a variety of ways, including the production of toxins by their own metabolism [7], the activation of inflammatory pathways by interaction with host cells [8, 9], and the destruction of intestinal protective barrier and immune system [10].

In recent years, although great progress has been made in the treatment and drug development of CRC [11, 12], there are still challenges in controlling efficiency, drug resistance, and side effects. Therefore, it is necessary to find safe and effective chemoprevention as well as treatment drugs without adverse reactions. Studies have shown that BBR, a drug derived from natural products, effectively reduces the recurrence risk of colorectal adenoma and polypoid lesions with mild damage to normal intestinal mucosal, implicating its potential usage as an anti-CRC drug [13].

**Fig. 1:**
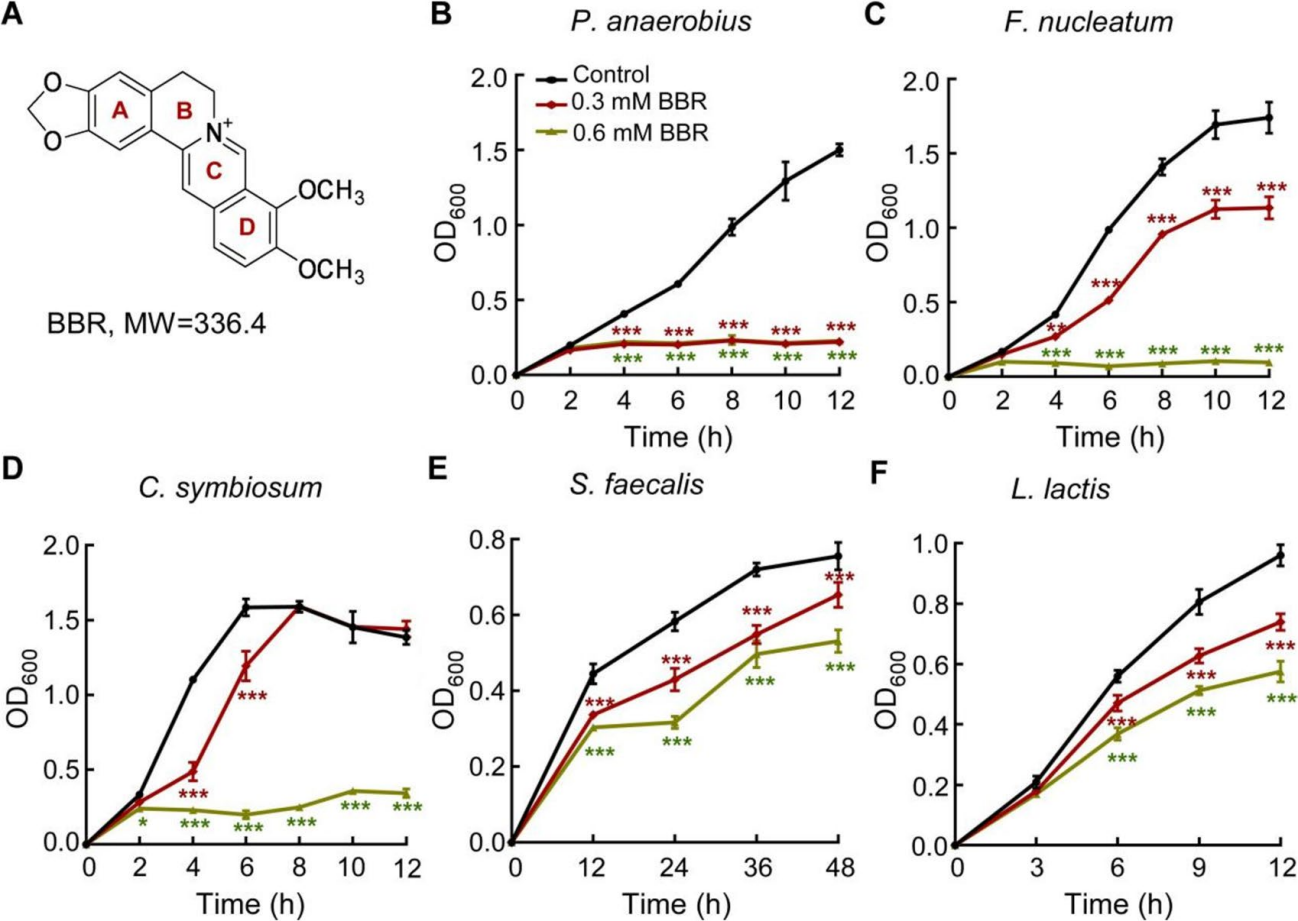
BBR inhibits the growth of pathogenic bacteria of CRC. **A** Chemical structure of BBR. **B**–**F** BBR inhibits the growth of pathogenic bacteria of CRC in a dose-dependent manner, including *P. anaerobius* ATCC 27337, *F. nucleatum* ATCC 10953, *C. symbiosum* ATCC 14940, *S. faecalis* ATCC 19433, and *L. lactis* DSM 20481. Data are presented as the mean ± S.D. of three independent replicates. The statistical significance of the differences in bacterial growth between the control strain and the other two strains (0.3 mM and 0.6 mM BBR) were assessed by the two-way ANOVA with Tukey’s multiple comparison test (***p < 0.001; **p < 0.01; *p < 0.05)

BBR is an isoquinoline alkaloid drug isolated from the plant *Coptis chinensis* and used commonly to treat diarrhea as a nonprescription drug (Fig. 1A). Recently, it was discovered that BBR exhibits potent anti-cancer activity [13–17]. For CRC, BBR inhibits proliferation, invasion, and metastasis of CRC cells by downregulating the COX-2/PGE2-JAK2/STAT3 signaling pathway [18], the β-catenin signaling pathway [19], and the mammalian target of rapamycin (mTOR) pathway [20]. The nuclear receptor retinoid X receptor α (RXRα) and adenosine monophosphate-activated protein kinase (AMPK) are potential direct molecular targets of BBR in CRC cells [19, 20]. Besides the direct action on cancer cells, BBR also slows tumor progression by modulating gut microbiota. Intestinal flora is inextricably related to the occurrence and development of CRC, and various CRC pathogenic bacteria have been used as important predisposing factors. Recent studies showed that BBR significantly increases the abundance of short-chain fatty acids (SCFA)-producing beneficial bacteria and reduces the abundance of opportunistic pathogens [21, 22], but the direct molecular target of BBR in pathogenic bacteria and its mechanism of action are not clear.

Therefore, in this study, we explored the action target and molecular mechanisms of BBR against the occurrence and development of CRC from the perspective of intestinal microorganisms. We first showed that BBR inhibits the growth of pathogenic bacteria related to CRC. By using biotin-conjugated BBR, we performed the affinity-based target profiling of BBR and discovered that formate tetrahydrofolate ligase (FtfL) is the direct target of BBR in *P. anaerobius*. Further biochemical results showed that BBR exhibits potent inhibitory effect on FtfL enzymatic activity. We subsequently determined the crystal structure of *Pa*FtfL complexed with BBR and found that BBR binds at both the “allosteric site” and “active site” of *Pa*FtfL and thereby likely inhibits *Pa*FtfL via interfering conformational flexibility of *Pa*FtfL tetramer and competing with its substrate ATP. In addition, BBR also inhibits the enzymatic activities of FtfL homologous in human tumor cells. Thus, BBR interferes with intestinal pathogenic bacteria by targeting FtfLs, providing a basis for BBR to play a new anticancer role in clinical practice.

## Results

### Inhibitory effect of BBR on pathogenic bacteria of CRC

To investigate the effect of BBR on intestinal microbes associated with CRC, we first tested the inhibitory activity of BBR on five selected pathogenic bacteria, *Peptostreptococcus anaerobius* ATCC 27337, *Fusobacterium nucleatum* ATCC 10953, *Clostridium symbiosum* ATCC 14940, *Streptococcus faecalis* ATCC 19433, and *Lactococcus lactis* DSM 20481, which were reported to cause disease by interacting with host cancer cells to either activate inflammatory pathways [8, 9] or promote cell secretion of virulence factors [7]. The results showed that BBR had differential dose-dependent inhibition on the five pathogenic bacteria at the intestine dose of BBR upon oral administration [23] (Fig. 1B–F), among which *P. anaerobius* was the most sensitive to BBR, followed by *F. nucleatum*.

For comparison, we also tested the effect of BBR on five reported probiotics, *Lactobacillus acidophilus* ATCC 4356, *Lactobacillus reuteri* DSM 17938, *Lactobacillus plantarum* DSM 13171, *Lactobacillus fermentum* CGMCC 1.1880, and *Lactobacillus bulgaricus* BD 15. Based on the growth phenotype, BBR promotes *L. acidophilus*; shows little effect on *L. reuteri*, *L. plantarum*, and *L. fermentum*; and inhibits *L. bulgaricus* to some extent (Additional file 1: Figs. S1A-E). These results indicate that BBR inhibits the growth of intestinal CRC-driving bacteria, while having much less inhibitory effect on beneficial bacteria in general.

### BBR targets *Pa*FtfL and inhibits the enzyme activity

To identify the direct molecular target of BBR in these CRC-driving bacteria, we adopted the activity-based protein profiling (ABPP) strategy that has been widely used in the discovery of drug targets. We first synthesized a BBR-biotinylated probe (BBP) comprising BBR, a C-9 hydrophilic linker, and biotin as reported [24] (Additional file 1: Fig. S2). Subsequently, BBP was incubated with cell lysate of *P. anaerobius* (the aforementioned pathogen with the highest sensitivity to BBR) to allow the binding of its potential target proteins.

**Fig. 2:**
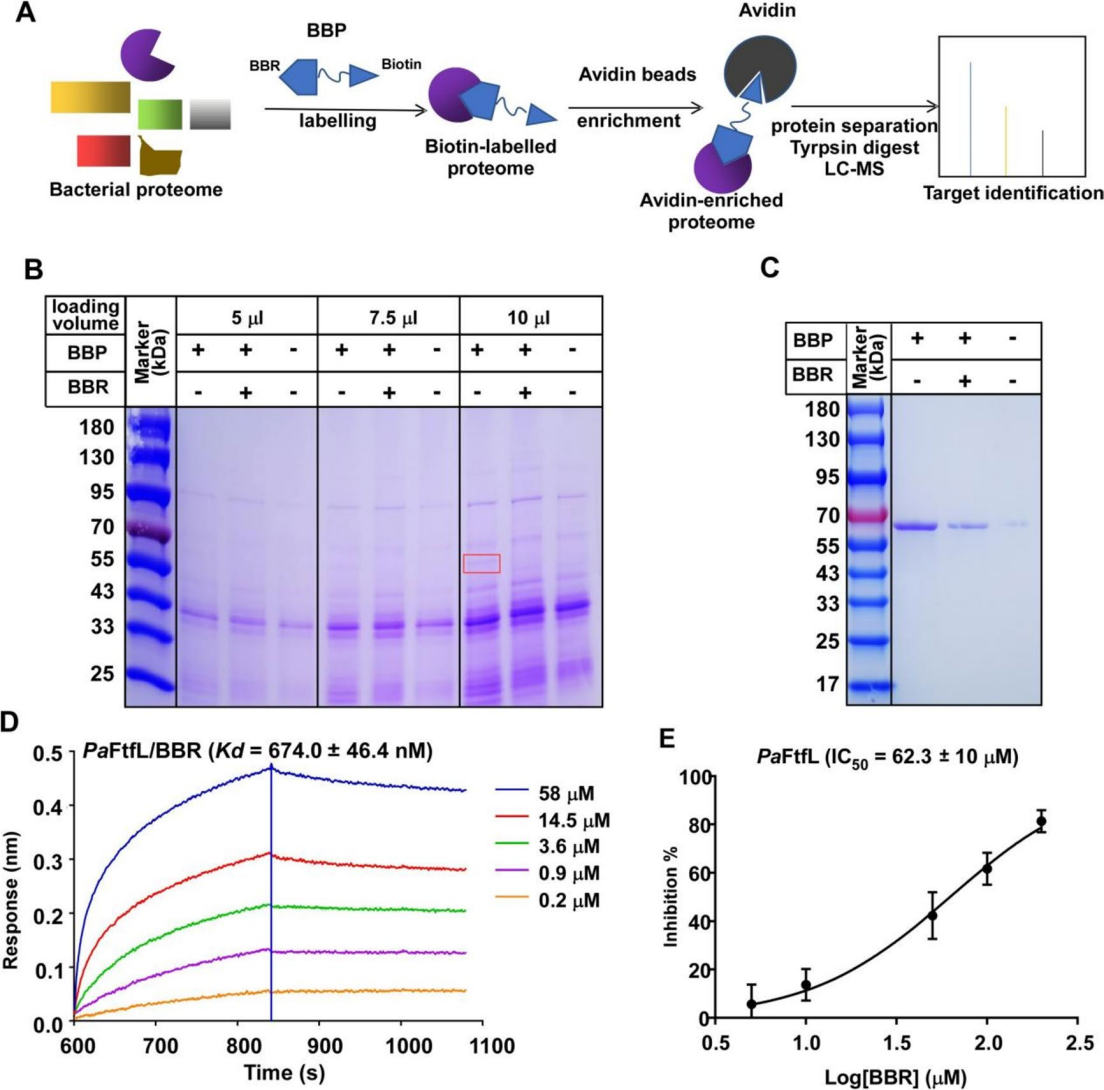
BBR directly binds to *Pa*FtfL with high affinity. **A** The scheme of target identification of BBR in *P. anaerobius* using probe-based target identification strategy. **B** BBR-biotinylated probe (BBP) specifically binds to a 60-kDa protein. BBP (100 μM) was incubated with cell lysate of *P. anaerobius* with or without BBR (1 mM); the SDS-PAGE analysis of probe-bound proteins showed a band of about 60 kDa specific to BBR (red box). The identities of the band were determined by LC–MS. **C** The pull-down assay show BBR binds with *Pa*FtfL, using purified *Pa*FtfL (3 μM) and BBP; BBP (100 μM) was incubated with *Pa*FtfL with or without BBR (1 mM). **D** BBR directly binds *Pa*FtfL. The kinetics 1:1 binding model was applied for fitting the data to determine the binding affinity. **E** Effect of BBR on *Pa*FtfL enzymatic activity. The dose-dependent inhibitory curve was fit to calculate the IC_50_. Data shown are mean ± S.D. from three independent experiments. The one-way ANOVA test was used for statistical analysis, *p* < 0.01

BBP enriched a protein band of ~ 60 KDa on the SDS-PAGE, and this enrichment was disrupted by incubation of excessive unmodified BBR, indicating that the target protein was specifically enriched by the BBR moiety of BBP (Fig. 2B). After digestion with trypsin, the proteins captured by BBP were analyzed by liquid chromatography-tandem mass spectrometry (LC–MS), and a total of 71 proteins were detected, of which 8 proteins that hitted with better BBR competitive effect were selected based on the intensity ratio of the hits between the experimental group and competitive group (Additional file 1: Table S3). Further results of the pull-down assay narrowed onto two proteins, FtfL (formate tetrahydrofolate ligase) and EF4 (elongation factor 4) (Fig. 2C and Additional file 1: Fig. S3). Next, we measured the binding affinity of BBR with *Pa*FtfL and *Pa*EF4 and confirmed the direct binding of *Pa*FtfL with BBR (*Kd* = 674 nM) by the bio-layer interferometry (BLI) method (Fig. 2D). *Pa*EF4 showed non-detectable binding affinity to BBR by BLI and thus was not discussed in this study (Additional file 1: Fig. S4). This may be caused by the imprecise quantification of the pull-down experiments or non-specific binding. In short, we identified that FtfL is the direct binding target of BBR in *P. anaerobius.*

FtfL is a key enzyme in C1 metabolism pathway. It catalyzes the synthesis of formyltetrahydrofolic acid from formic acid and tetrahydrofolic acid. Formyltetrahydrofolic acid is then converted through two reactions to 5,10-methylene tetrahydrofolic acid [25], a metabolite for biosynthesis of pyrimidines and amino acids [26]. In order to explore whether BBR binding affects the enzymatic activity of *Pa*FtfL, we incubated *Pa*FtfL with different concentrations of BBR (5, 10, 50, 100, 200 μM) and determined its enzyme activity. The results showed that BBR significantly inhibited the activity of *Pa*FtfL in a dose-dependent manner with an IC_50_ of 62 μM (Fig. 2E). Thus, we showed that BBR directly inhibits the enzymatic activity of FtfL in intestinal cancer pathogens.

### Structural basis and molecular mechanism of BBR inhibiting FtfL

**Fig. 3:**
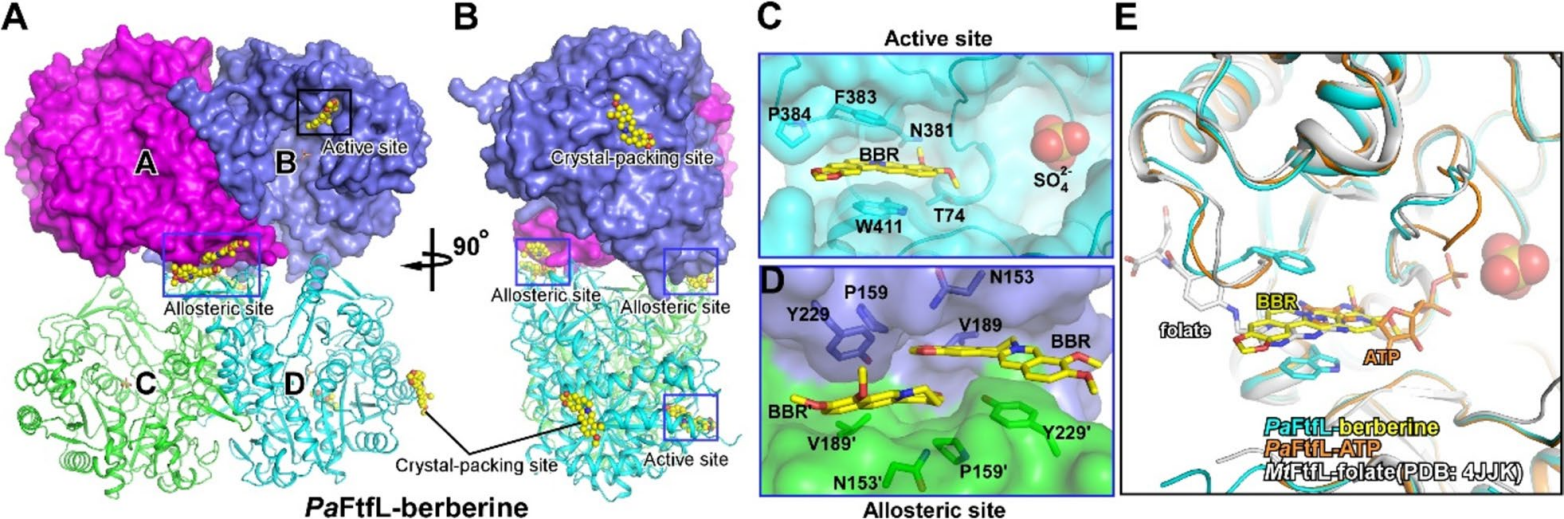
The overall structure of *Pa*FtfL complexed with BBR. **A**, **B** The overall structure of *Pa*FtfL-BBR. The four protomers are in different colors. The top dimer is in surface presentation and the bottom dimer is in cartoon presentation. The three types of BBR binding sites are labeled. **C** The interaction between *Pa*FtfL and BBR in the active site. **D** The interaction between *Pa*FtfL and BBR in the allosteric site. **E** Structural comparison among crystal structures of *Pa*FtfL-BBR, *Pa*FtfL-ATP, and *Mt*FtfL-folate (PDB:4JJK)

To further investigate the structural mechanism of FtfL inhibition by BBR, we determined the crystal structures of *Pa*FtfL apo enzyme, *Pa*FtfL complexed with its substrate ATP, as well as *Pa*FtfL complexed with BBR at 2.0 Å, 2.3 Å, and 2.6 Å resolutions, respectively (Additional file 1: Table S4). *Pa*FtfL exhibits a tetramer in all three crystal structures, consistent with other reported FtfL crystal structures (Fig. 3A, B, and Additional file 1: Figs. S5A and S5B) [25, 27]. Protomers A/B and protomers C/D form two stable dimers with an interface area of 2100 Å^2^, and the two dimers assemble into a tetramer with an additional interface area of 620 Å^2^. Each protomer comprises its own solvent-exposed active site close to the protomer interface. Structural comparison reveals that *Pa*FtfL adopts essentially the same overall fold for each of the protomers and exhibits an identical active site as in reported crystal structures of FtfL from other bacterial species (Additional file 1: Figs. S5C and S5D), indicative its conserved catalytic mechanism.

The crystal structures show that BBR inhibits the enzymatic activity of FtfL likely through direct competition with substrate and allosteric inhibition of catalytic reaction. In the crystal structure of *Pa*FtfL-BBR, the Fo-Fc electron density difference map clearly shows three types of binding sites of BBR (Additional file 1: Figs. S5E, S5F and S5I). But we suspect that only two of them are related to inhibitory function. In the first type of binding site, named as “active site,” two molecules of BBR occupy the active site of protomers B and D, respectively, in which BBR mainly makes Van der Waals interactions with surrounding residues (T74, N381, F383, P384, W411) (Fig. 3C). Specifically, the B ring of BBR is sandwiched between the aromatic side chains of residues F383 and W411 (Fig. 3C). Structural comparison among our crystal structures of *Pa*FtfL-BBR, *Pa*FtfL-ATP, and *Mt*FtfL-folate suggests that BBR overlaps with ATP and tetrahydrofolate on binding to FtfL (Fig. 3E and Additional file 1: Fig. S5G), indicating that BBR likely competes with ATP and tetrahydrofolate to bind FtfL active site. The biochemical assay also showed that the apparent Michaelis constants (*K*_m_) of ATP and tetrahydrofolate increased when BBR was added (Additional file 1: Fig. S6, Table S5), supporting that BBR inhibits the enzymatic activity of FtfL partially through direct competition.

In the second type of binding site, named as “allosteric site,” four BBR molecules bind at the dimer-dimer interface, where two BBR molecules occupy the interface of protomers A/D and another two BBR molecules occupy the interface of protomers B/C. In each of the two allosteric sites, two molecules of BBR stack together in a face-to-face fashion with their 2,3-methylenedioxy ring, while the D ring of the two BBR rotates ~ 90° away from each other along the axis perpendicular to the BBR plain and makes π-π stacking interactions with residue Y229 (Fig. 3D). Each BBR molecule also makes Van der Waals interactions with the side-chain atoms of surrounding residues (N153, P159, V189) (Fig. 3D). The presence of BBR in the protomer interface causes a 4.4° rotation of one dimer towards the other (Additional file 1: Fig. S5H). We show that the protomer interface allosterically affects the active center, as alanine substitution of the interface residue Y229 reduced the *Pa*FtfL activity by 63% (Additional file 1: Fig. S7). Moreover, the disruption of both allosteric and active sites completely abolished the enzymatic activity of *Pa*FtfL (Additional file 1: Fig. S7). We infer that the binding of BBR at the subunit interface might contribute to its inhibition on *Pa*FtfL catalytic reaction through allosteric effect, and simultaneous binding at both allosteric and active sites explains the potent inhibition of BBR on *Pa*FtfL.

To validate the interactions of BBR and *Pa*FtfL observed in our crystal structure, we measured the binding affinity of BBR with *Pa*FtfL derivatives bearing alanine substitutions at its two binding sites (“active site” and “allosteric site”) by the BLI assay. The results show that disrupting the “active site” significantly reduces BBR binding, as evidenced by the 6- and 34-fold reduction of binding affinity of W411A and F383A, respectively (Additional file 1: Table S6), and disruption of the “allosteric site” (Y229A) also substantially reduces the binding affinity of BBR (Additional file 1: Table S6).

### Distribution and abundance of FtfL in microbial communities and human microbiome

**Fig. 4:**
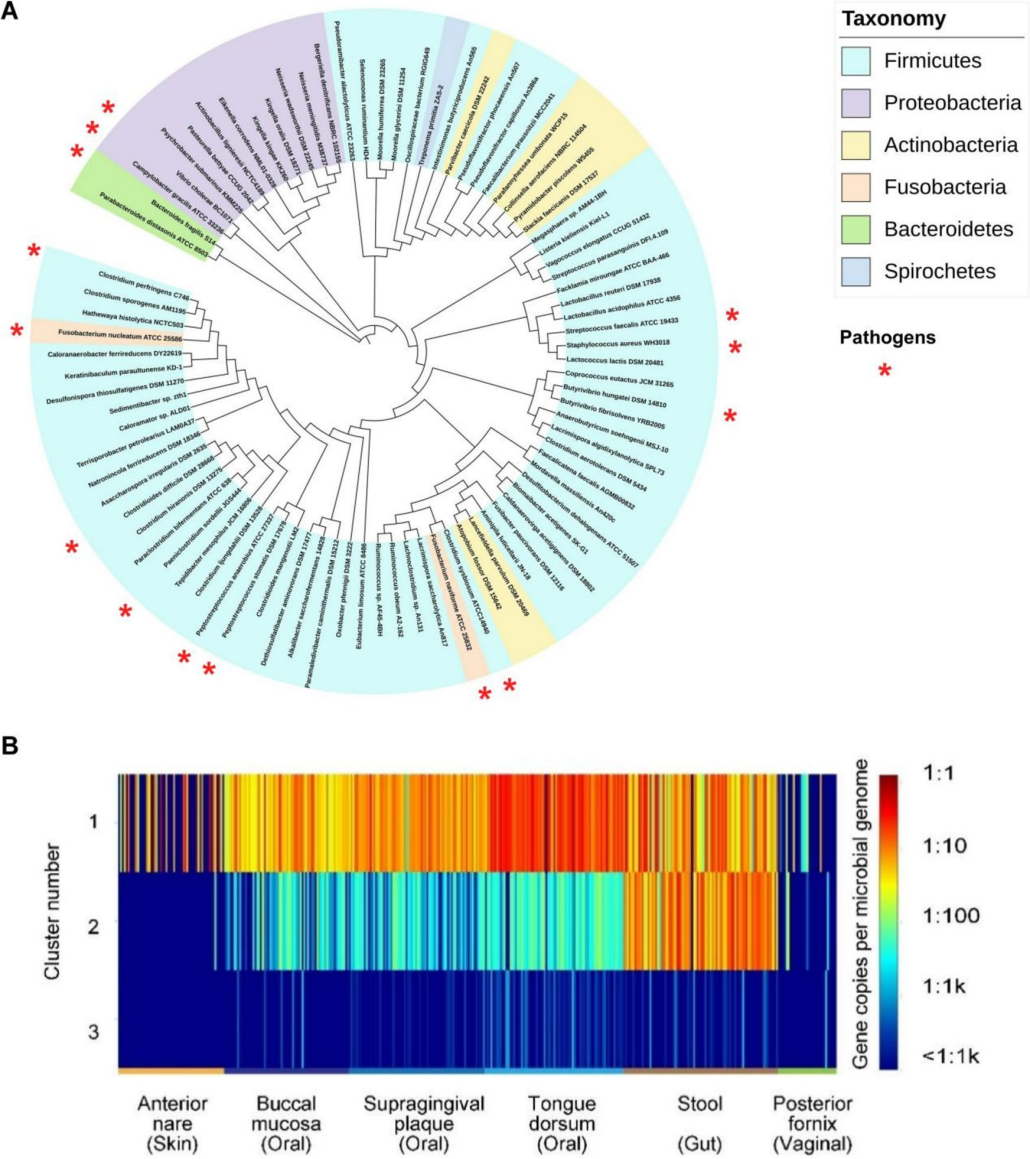
The distribution and abundance of FtfL in microbial communities and human microbiome. **A** Phylogenetic analysis of FtfLs. The maximum-likelihood phylogenetic tree was constructed that based on the protein sequences of putative FtfL enzymes from Firmicutes, *Fusobacteria*, *Bacteroidetes*, *Actinobacteria*, *Proteobacteria*, and *Spirochetes*. Black circles on branches indicate bootstrap values greater than 0.7 from 300 bootstrap replicates. The human pathogens strains are highlighted with red asterisk. **B** Heatmap of the abundance and distribution of three FtfL clusters

To investigate the distribution of FtfL proteins in microbial communities, we used TBLASTN tool to search NCBI database for *Pa*FtfL homologs and then performed maximum likelihood phylogenetic analysis based on the non-redundant hits with coverage greater than 90% and similarity greater than 50%. The results showed that FtfL is widely distributed across the bacterial kingdom. Among them, the intestinal dominant bacterial group *Firmicutes* accounted for the largest proportion (Fig. 4A). FtfL is also found in certain bacterial species of *Fusobacteria*, *Bacteroidetes*, *Actinobacteria*, *Proteobacteria*, and *Spirochetes*. For pathogenic microorganisms, in addition to CRC-driving bacteria mentioned above, FtfL is also present in the pathogens of enteritis, such as *Clostridium perfringens*, *Clostridium difficile*, *Vibrio cholerae*, *Campylobacter gracilis*, *Bacteroides fragilis*, and other disease-causing bacteria, such as *Staphylococcus aureus* and *Peptostreptococcus stomatis* (Fig. 4A).

To further understand the abundance of FtfL in human microorganisms, we first clustered the FtfL homologous sequences in the above phylogenetic tree by protein sequence similarity network (SSN) analysis and divided them into three different clusters. Then, metagenomic data from 380 samples of healthy participants in the ShortBRED database were used to analyze the distribution and abundance of each cluster of FtfL in the human microbiota (Fig. 4B). The data were obtained from six different parts of the body: feces, buccal mucosa, supragingival plaque, dorsum linguae, anterior naris, and vaginal fornix in relation to aerobic (skin), microaerobic (mouth and vagina), and anaerobic (gut) environments. All the three clusters of FtfL were detected in the human microbiome, but the distribution and abundance of each cluster were different. Among these, cluster 1 contained 75 FtfL enzymes (including *Pa*FtfL in *P. anaerobius*), which were abundant in samples from all human parts, especially fecal and oral samples. The abundance of these genes ranged from 0.01(1 copy per 100 cells) to 0.1(1 copy per 10 cells). Cluster 2 (*Lactobacillus acidophilus* and *Lactobacillus reuteri*) was mainly distributed in fecal samples with moderate abundance; cluster 3 (*Parabacteroides distasonis* and *Bacteroides fragilis*) mainly existed in oral and fecal samples, but the abundance was relatively low. Therefore, FtfL is widely distributed in human microorganisms with different abundances, which is presumed to be related to the physiological environment of the host.

### Effects of BBR on FtfLs from different intestinal bacteria

In order to understand how BBR acts on FtfLs of different intestinal bacteria, four strains each of CRC-driving bacteria, enteritis pathogenic bacteria, non-pathogenic bacteria, and probiotics were selected respectively from the above phylogenetic tree as representatives for testing (Table 1). The results showed that BBR had strong concentration-dependent inhibition on the activities of FtfLs derived from three CRC pathogens: *F. nucleatum*, *C. symbiosum*, and *L. Lactis* (Table 1 and Additional file 1: Fig. S8). Among enteritis pathogens, FtfLs from *B. fragilis*, *C. difficile*, *V. cholerae*, and *C. perfringens* were inhibited by BBR (Table 1 and Additional file 1: Fig. S8). For non-pathogenic bacteria, BBR had no significant effect on enzymatic activity of FtfL from *Clostridium sporogenes*, the activity of FtfL from *Parabacteroides distasonis* was slightly inhibited, and the activities of FtfLs from *Clostridium ljungdahlii* and *Ruminococcus obeum* was significantly inhibited (Table 1 and Additional file 1: Fig. S8); FtfL from *L. plantarum* in the probiotics was slightly inhibited by BBR (Table 1 and Additional file 1: Fig. S8). The enzymatic activities were not detected in FtfLs derived from CRC-causing bacterium *S. faecalis* and three probiotic strains, *L. reuteri*, *L. acidophilus*, and *L. fermentum*, so further targeted inhibition testing was not performed.

**Table 1:** Effect of BBR on FtfLs from different strains. The inhibition of BBR on FtfLs from *S. faecalis*, *L. reuteri*, *L. acidophilus*, *and L. fermentum* is not determined due to non-detectable enzymatic activity; “ns” means no significance, representing that FtfL from this strain is not inhibited by BBR

Strain	*ftfl* Gene ID	Inhibited by BBR (IC_50_)
**CRC pathogens**		
*Peptostreptococcus anaerobius*	79,842,341	62.3 ± 10 μM
*Fusobacterium nucleatum*	45,633,862	106.2 ± 27.0 μM
*Clostridium symbiosum*	57,969,199	128.9 ± 17.1 μM
*Lactococcus lactis*	69,713,002	118.1 ± 18.5 μM
*Streptococcus faecalis*	60,894,021	/
**Colitis pathogens**		
*Bacteroides fragilis*	66,328,797	105.1 ± 69.8 μM
*Clostridioides difficile*	66,353,223	158.0 ± 68.7 μM
*Vibrio cholerae*	69,721,326	129.6 ± 12.3 μM
*Clostridium perfringens*	69,450,453	104.9 ± 3.15 μM
**Nonpathogenic bacteria**		
*Clostridium ljungdahlii*	45,181,033	83.8 ± 10 μM
*Clostridium sporogenes*	69,424,169	ns
*Parabacteroides distasonis*	57,237,971	> 200.0 μM
*Ruminococcus obeum*	15,206,023	100.1 ± 3.26 μM
**Probiotics**		
*Lactobacillus plantarum*	57,025,392	> 200.0 μM
*Lactobacillus reuteri*	69,707,752	**/**
*Lactobacillus acidophilus*	56,943,130	**/**
*Lactobacillus fermentum*	12,456,081	**/**

In summary, we showed that BBR exhibits a similar inhibitory effect on FtfLs in the tested CRC-driving pathogens and enteritis pathogenic bacteria and relatively little effect on those probiotics. The result is consistent with the finding that BBR inhibits the growth of CRC pathogenic bacteria but not of probiotics and suggests that FtfL is likely the primary target of BBR in pathogenic bacteria.

### FtfL homologs in human cells are also bound by BBR

**Fig. 5:**
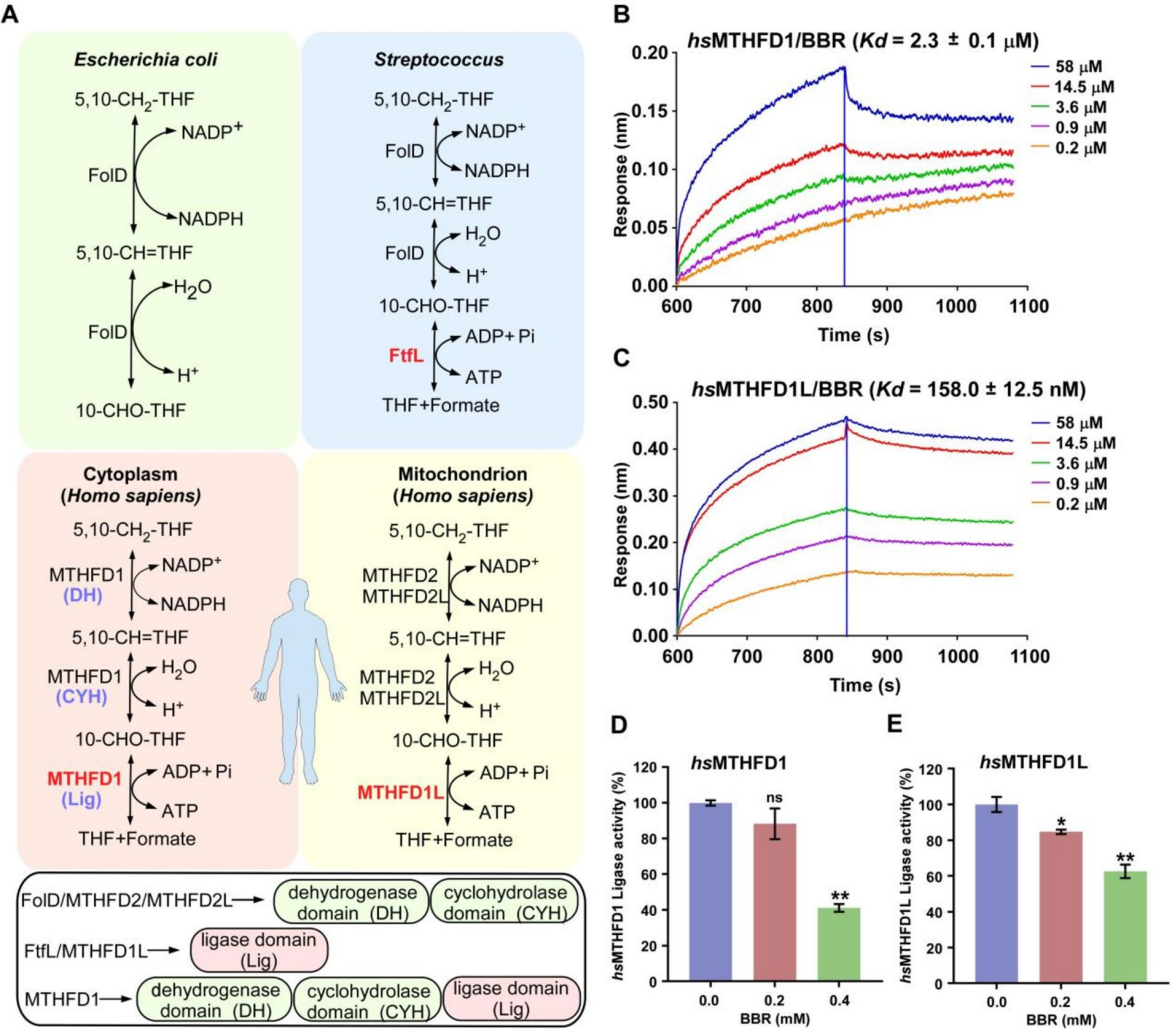
BBR binds with the human enzymes *hs*MTHFD1 and *hs*MTHFD1L. **A** The proteins with the activity of formate-THF ligase in different organisms. In *E. coli*, there is no protein with the activity of formate-THF ligase, while in several other bacteria such as *Streptococci*, FtfL displays the activity of formate-THF ligase. In humans, there are different enzymes in the cytoplasm and the mitochondrion, respectively. In the cytoplasm, MTHFD1 exerts formate-THF ligase, 5,10-methenyl-THF cyclohydrolase, and 5,10-methylene-THF dehydrogenase functions. In the mitochondrion, MTHFD1L plays the same role as FtfL. **B**, **C** BBR directly binds *hs*MTHFD1 and *hs*MTHFD1L. The binding affinities (*Kd*) were determined by fitting the binding data to a kinetics 1:1 binding model. **D**, **E** Effect of BBR on *hs*MTHFD1 and *hs*MTHFD1L enzymatic activities. Data are presented as the mean ± S.D. of three independent replicates. The one-way ANOVA with Tukey’s multiple comparison test was used for statistical analysis, *p < 0.05, **p < 0.01, ***p < 0.001

Protein homology analysis showed that FtfL homologs also exist in human cells, including MTHFD1 (methylenetetrahydrofolate dehydrogenase 1) and MTHFD1L (methylenetetrahydrofolate dehydrogenase 1-like). They are highly expressed in many tumor cells (Additional file 1: Fig. S9) and are potential clinical therapeutic targets as reported [28, 29]. MTHFD1 is a cytosolic trifunctional protein that catalyzes a three-step reaction from formate and tetrahydrofolate to 5,10-methylene tetrahydrofolic acid; MTHFD1L is a monofunctional mitochondrial protein with formate-THF ligase activity (Fig. 5A). They are key enzymes in one-carbon metabolism, related to nucleic acid and protein synthesis, DNA methylation, and repair, and play an important role in human body [29].

Human MTHFD1 and MTHFD1L share 43.4% and 42.8% sequence identity with microbial *Pa*FtfL respectively, and intriguingly the key residues around the BBR binding sites are conserved (Additional file 1: Fig. S10). We are intrigued to know whether they could also be targeted by BBR. To answer this question, we purified these two proteins and measured the binding affinity of BBR by bio-layer interferometry (BLI). The results showed that BBR binds to both MTHFD1 and MTHFD1L in a dose-dependent manner with *Kd* values of 2.3 μM and 158 nM, respectively (Fig. 5B and C). Next, we examined whether such interaction leads to enzyme inhibition and found that BBR inhibited the ligase activities of both MTHFD1 and MTHFD1L in a concentration-dependent manner. When the concentration of BBR reached 0.4 mM, their enzyme activities decreased by 58% and 36%, respectively (Fig. 5D and E). In short, our data show that BBR has moderate inhibitory activity towards human MTHFD1 and MTHFD1L in vitro. However, it remains to be determined whether such an inhibition plays any role in the anti-tumor activity of BBR [14].

## Discussion

We discovered that formate tetrahydrofolate ligase (FtfL) is a direct target of the natural product medicine BBR. The drug inhibits the enzymatic activity of FtfLs in various intestinal pathogenic bacteria (especially *P. anaerobius*) to weaken their viability, thereby preventing and reducing the occurrence and development of CRC, forming the anti-cancer “BBR/FtfL” axis. We also showed that BBR exhibits certain selectivity on gut bacteria, with much higher inhibitory efficacy on gut pathogens. We further showed that the enzymatic activity of the FtfL homogenous proteins in human cells, MTHFD1 and MTHFD1L that are associated with CRC, is also moderately inhibited by BBR. This suggests that BBR may also play a role by directly targeting MTHFD1 and MTHFD1L in colorectal cancer cells.

The known direct targets of BBR include RXRα (retinoid X receptor α) [19], G4s (G quadruplexes) [30], UHRF1 (ubiquitin-like with PHD and RING finger domains) [31], and MAP2K7 (mitogen-activated protein kinase 7) [32] in human somatic cells, as well as FtsZ (filamentation temperature sensitive protein Z) in microorganisms [33]. BBR interacts with them to exert antitumor, anti-inflammatory, and antibacterial effects, respectively. In this study, we identified a novel target of BBR in CRC pathogens, FtfL, a key metabolic enzyme that is vital for bacterial growth. The elucidation of the FtfL-BBR interaction mechanism provides a new perspective for the prevention and control of CRC. However, FtsZ was not identified in our study; it is possible that biotin modification of BBP blocks the interaction with FtsZ.

Through structural analysis of *Pa*FtfL-BBR complex, we revealed the interaction sites of FtfL, namely “active site” and “allosteric site.” Comparison of the structures of enzyme–substrate complexes *Pa*FtfL-ATP and *Mt*FtfL-folic acid showed that the FtfL binding sites of both substrates significantly overlapped with the “active site” described above (Fig. 3E and Additional file 1: Fig. S5G), suggesting that BBR may inhibit FtfL activity by competing with ATP and tetrahydrofolic acid for binding to this site. On the other hand, the binding of BBR to the “allosteric site” of the tetramer interface of FtfL changes its conformation and limits the flexibility of the tetramer, thus affecting the activity of FtfL. It is difficult to determine which site BBR displays higher affinity at, but we infer that both sites contribute to the binding of BBR to FtfL, as disruption of either of the two binding sites impairs the interaction of BBR and FtfL. In addition, in the third type of binding site, named as “crystal-packing site,” two molecules of BBR locates between two crystal symmetry-related tetramers of FtfL, and thereby we infer that the interaction is not relevant to its inhibitory function (Additional file 1: Fig. S5I). Just as we suspected, the disruption “crystal-packing site” does not affect the enzymatic activity of *Pa*FtfL (Additional file 1: Fig. S7).

Phylogenetic analysis showed that FtfL and its homologs were widely present in human microorganisms, especially in pathogenic bacteria located in intestinal tract and oral cavity. This may partly explain why BBR has a wide range of effects on microorganisms and various pharmacological effects. Our data show that BBR displays certain selectivity on bacteria inhibition. It significantly inhibits several intestinal cancer pathogens but has little effect on the tested probiotics. This is consistent with the observation that BBR reduces the abundance of pathogenic bacteria and does not affect the abundance of probiotics in the treatment of diabetes [22, 34].

In short, the targeting effect of BBR is both pleiotropic and selective, which makes it a magical “versatile” medicinal function. BBR has been widely used in the treatment of intestinal infections. Clinical practice and research have shown that the drug is safe with a low incidence of side effects. The potential value of BBR as an antitumor drug in the prevention and treatment of tumor recurrence has been extensively recognized, which actively promotes its clinical use [13, 35, 36]. Its low cost and safety also make it possible for long-term use. Our discovery of FtfL as the molecular target of BBR in bacteria provides a mechanistic basis for its clinical efficacy in the treatment of malignant CRC and enteritis. Our study also implies that FtfL might serve as a new molecular target of bacteria in treating various human diseases by controlling the gut microbiome.

## Conclusions

In summary, this study identified the protein FtfL, a key enzyme in C1 metabolism, is the molecular target of BBR in *P. anaerobius*. BBR directly binds to FtfL to inhibit its enzyme activity. Based on this, the crystal structure of *Pa*FtfL-BBR complex elucidated the molecular mechanisms of BBR inhibition on FtfL. In addition, the enzymatic activities of FtfL homologous proteins in human tumor cells can also be inhibited by BBR. Our findings provide insights into the molecular mechanisms and target of BBR, which may further open its therapeutic applications in CRC treatment.

## Methods

### Chemicals and reagents

Commercial chemicals were obtained from Sigma-Aldrich (Sigma-Aldrich Co., St Louis, USA), Sangon (Sangon Biotech Co., Ltd., Shanghai, China), Weikeqi (Weikeqi Biological Technology Co., Ltd., Sichuan, China), and Sinopharm (Sinopharm Chemical Reagent Co., Ltd., Shanghai, China). BBR chloride was dissolved in DMSO to obtain stock solution (60 mM). THF was dissolved in 2-mercaptoethanol (1.0 M) to generate stock solution (10 mM, neutralized with 1 M KOH). ATP was dissolved in Tris–HCl (100 mM, pH = 7.9) to obtain stock solution (10 mM). PBS was prepared as follows: 29.22 g (500 mM) NaCl, 0.20 g (2.7 mM) KCl, 1.44 g (10.1 mM) Na_2_HPO_4_, and 0.24 g (1.8 mM) KH_2_PO_4_ were dissolved in 1 L of double distilled water (pH = 7.0).

### Bacterial strains and culture conditions

*E. coli* DH5α and BL21 (DE3) as well as their derived strains were grown in LB (lysogeny broth) medium [37], with the addition of kanamycin (100 μg/mL) when needed. The *E. coli* DH5α and *E. coli* BL21 (DE3) strains were used for gene cloning and protein expression, respectively.

*Peptostreptococcus anaerobius* ATCC27337, *Fusobacterium nucleatum* ATCC10953, and *Clostridium symbiosum* ATCC14940 were grown in the brain heart infusion (BHI) broth supplemented with hemin, K_2_HPO_4_, vitamin K_1_, and l-cysteine [38]. *Streptococcus faecalis* ATCC19433, *Lactococcus lactis* DSM20481, *Lactobacillus acidophilus* ATCC4356, *Lactobacillus reuteri* DSM17938, *Lactobacillus plantarum* DSM13171, *Lactobacillus fermentum* CGMCC1.1880, and *Lactobacillus bulgaricus* BD15 were grown in the MRS medium [39]. Anaerobic bacteria are cultured in an anaerobic chamber (Whitley A35 Anaerobic Workstation, Don Whitley Scientific Limited, Bingley, West Yorkshire, UK).

### Synthesis of the BBR-biotinylated probe

The BBR-biotinylated probe (BBP, 4), consisting of BBR, biotin, and C-9 hydrophilic linker, was synthesized as described previously (Fig. S2) [24]. In brief, the compound 1 (1.006 mmol BBR) dissolved in DMF (10 mL) was mixed with propargyl bromide (2.415 mmol). The mixture was stirred and then recrystallized from diethyl ether to generate compound 2 as a brown solid. Next, in the presence of CuSO_4_ and sodium ascorbate, BBP (4) was obtained from compound 2 and azide biotin (3) through click chemical reaction. Finally, the solvent was concentrated, and the residues were purified to obtain BBP (C_40_H_50_N_7_O_9_S^+^).

### Identification of BBR-binding proteins

*P. anaerobius* ATCC27337 was grown in the BHIS medium under anaerobic conditions. The cells were collected by centrifugation (8000 × *g*; 5 min; 4 °C) at an OD_600_ of 1.0. Cells were resuspended in lysis buffer (PBS + 1 mM PMSF) and then lysed using a cell disruptor (French Press, Constant Systems Limited, Northants, UK). The lysate was centrifugated at 15,000 × *g* for 60 min at 4 °C. The supernatant (containing 200 μg of total protein) was incubated with BBP (100 μM) for 2 h at room temperature. The mixture was added to streptavidin agarose resin and then incubated for 0.5 h at room temperature on a rotating apparatus. After five times of washing with lysis buffer (PBS + 0.05% Tween 20 + 1% PEG, pH 7.0) and centrifugation (1000 × *g* at 25 °C for 10 s) to remove unbound components, the BBP-bound proteins in streptavidin agarose resin were loaded on a 12% SDS-PAGE gel and then stained by Coomassie Blue. The specific bands of interest were cut out and trypsinized overnight. The extracted peptides were identified by LC–MS (Orbitrap™ mass analyzer, Thermo Fisher Scientific, Vilnius, Lithuania). Here, the following controls were set: (i) the aforementioned supernatant (containing 200 μg of total protein) was mixed with BBP (100 μM) and free BBR (1 mM); (ii) the supernatant (containing 200 μg of total protein) was mixed with biotin (100 μM).

To validate BBR-binding protein, the recombinant target proteins (3 μM) were prepared and subjected to the same manipulations as described above. The following controls were set: (i) 3 μM recombinant target protein mixed with BBP (100 μM) and free BBR (1 mM); (ii) 3 μM recombinant target protein mixed with biotin (100 μM).

### Gel digestion and liquid chromatography-mass spectrometry analysis

The gel bands of interest were cut out from SDS-PAGE, destained with 30% ACN/100 mM NH_4_HCO_3_, and dried in a vacuum centrifuge. The in-gel proteins were reduced with dithiothreitol (10 mM DTT/100 mM NH_4_HCO_3_) for 30 min at 56 °C and then alkylated with iodoacetamide (200 mM IAA/100 mM NH_4_HCO_3_) in dark at room temperature for 30 min. Next, gel pieces were rinsed with 100 mM NH_4_HCO_3_ and ACN and then digested overnight with trypsin (12.5 ng/μl) in 25 mM NH_4_HCO_3_. The peptides were extracted with 60% ACN (containing 0.1% TFA), and pooled and dried completely by a vacuum centrifuge.

The LC–MS analysis was carried out on a Q Exactive mass spectrometer combined with an Easy HPLC system (Thermo Scientific, Vilnius, Lithuania). Each sample was loaded onto a reverse phase trap column linked to a C18-reversed phase analytical column and eluted with a flow rate of 0.3 μl/min for 30 min. The mobile phase A and B for HPLC separation were 0.1% formic acid in deionized water and 84% acetonitrile, respectively. The chromatography gradient was set up with the following linear increase: 5% to 35% of B within 22 min; 35% to 100% of B within 5 min; 100% of B for the last 3 min.

The mass spectrometer was operated in a positive ion mode with the following parameters: MS spectra in the range of 300–1800 m/z. Survey scans were acquired at a resolution of 70,000 at m/z 200, isolation width was 2 m/z, microscans to 1, and maximum inject time to 50 ms. Normalized collision energy was 27 eV, and the underfill ratio was defined as 0.1%. The instrument was run with peptide recognition mode enabled.

The Mascot 2.2 search engine (Matrix Science Ltd., London, UK) was used for protein identification. Searches of the MS data were performed based on the *P. anaerobius* UniProt database.

### Plasmid construction and site-directed mutagenesis

All the primers and plasmids used in this study were listed in Tables S1 and S2 respectively. The vector of pET28a-*Pa*FtfL for expressing the *ftfl* gene from *P. anaerobius* ATCC27337 was constructed as follows. In brief, the DNA fragment of *Paftfl* was obtained by PCR amplification using the primers *Paftfl*-F/R and the genomic DNA of *P. anaerobius* ATCC27337 as the template. And the DNA fragment was then inserted into the pET28a plasmid (digested with BamHI and NdeI) using a ClonExpress MultiS One Step cloning kit (Vazyme, Nanjing, China). The plasmids for expressing the other *ftfl* genes were constructed via the same steps.

The vectors pET28a-*Pa*FtfLY229A for expressing the mutated *ftfl* gene of *P. anaerobius* ATCC27337 was constructed as follows. In brief, the DNA fragment of the mutated *ftfl* gene was obtained by PCR amplification using the vector pET28a-*Pa*FtfL as the template and the primers *Paftfl*Y229A-F/R. The PCR product was treated with DPN1 to remove the original methylated plasmids and then transformed into *E. coli* DH5α. The vectors expressing the other mutated *ftfl* genes were constructed via the same steps.

### Gene expression and protein purification

The proteins used in this study were expressed in the *E. coli* BL21 (DE3) strain. Protein expression was induced with 0.3 mM IPTG at 16 °C when the cell density reached OD_600_ of 0.6 ~ 0.8. After 16 h of induction, cells were collected by centrifugation (12,000 × *g* for 5 min at 4 °C) and then resuspended in a lysis buffer (100 mM Tris–HCl, pH 7.9, 100 mM NaCl) with 1 mM PMSF. Cells were broken by using a cell disruptor (French Press, Constant Systems Limited, UK), and the lysate was centrifuged (15,000 × *g* for 60 min at 4 °C). The supernatant was loaded onto a Ni^2+^ Sepharose™ 6 fast flow agarose resin (GE Healthcare, Waukesha, WI, USA). The resin was then washed with lysis buffer (containing 15 mM, 30 mM imidazole) for the removal of non-target proteins, and eluted with lysis buffer (containing 300 mM imidazole). The eluted fractions were freed from imidazole by an Amicon Ultra 15 Centrifugal Filter (Millipore Billerica MA) with lysis buffer. Finally, the purified proteins were identified by SDS-PAGE and then concentrated (Amicon Ultra-4, Millipore, USA) and stored at − 80 °C.

### Bio-layer interferometry (BLI) assay

The interactions between BBR and the *Pa*FtfL (including its variants), *hs*MTHFD1, and *hs*MTHFD1L proteins were determined by using the ForteBio Octet RED 96 platform (Forte Bio, San Francisco, USA). A streptavidin matrix-coated sensor chip (SA chip) was firstly equilibrated with buffer A (lysis buffer with 0.05% Tween 20) followed with the immobilization BBP (200 μM) on the SA chip. Next, proteins with increasing concentrations (0.23, 0.91, 3.63, 14.50, and 58.00 μM) were passed on to the chip for the measurement of changes in response unit (nm). The program comprises the stabilization of the baseline with the buffer A for 1 min, 6 min incubation of the BBP with the SA chip for immobilization, stabilizing the baseline again for 3 min, association enabling interaction between proteins and compounds for 4 min, and dissociation for 4 min followed by a regeneration step. The interaction between BBR and the *Pa*EF4 protein (58 μM) was determined by using the same method. Raw data were pre-processed, analyzed, and fitted using the 1:1 binding model in the manufacturer’s software (9.0, Pall ForteBio Corp, Menlo Park, CA, USA) to generate kinetic parameters.

### Determination of inhibitory activity assay of BBR to FtfL

The activities of the FtfL enzymes were measured according to the protocol described previously [26].

The assay of the BBR’s inhibition on FtfLs was carried out as follows. In brief, 20 nM FtfLs (100 nM for LpFtfL because of its low activity) was preincubated with BBR (5, 10, 50, 100, and 200 μM) at 30 °C for 5 min. Then, the reaction was initiated by adding substrates (0.1 mM tetrahydrofolate, 2 mM MgCl_2_, 0.05 mM ATP, and 8 mM sodium formate). After 2 min of reaction, the reaction was terminated by adding HCl (0.36 M, 2 × volume of the reaction mixture).

The assay of the BBR’s inhibition on *hs*MTHFD1/*hs*MTHFD1L was performed as follows. Briefly, 400 nM *hs*MTHFD1 or *hs*MTHFD1L was mixed with BBR (200 or 400 μM) and preincubated at 30 °C for 5 min. Then, the reaction was initiated by adding substrates (2 mM tetrahydrofolate, 10 mM MgCl_2_, 40 mM sodium formate, and 5 mM ATP). After 2 min of incubation, the reaction was terminated by adding HCl (0.36 M, 2 × volume of the reaction mixture).

The product was detected with a maximum absorbance at 350 nm (*ε* = 24.9 mM^−1^ cm^−1^) using FLUOstar OPTIMA (BMG LABTECH, Offenburgh, Germany). The reaction was performed at 30 °C. The control reaction was performed by replacing BBR with DMSO. The half maximal inhibitory concentration (IC_50_) of BBR was adopted to represent the inhibitory efficiency of BBR on different FtfLs. Data analysis was performed in GraphPad Prism 7.0.

The enzyme kinetic constants of *Pa*FtfL were determined using two kinds of reaction mixtures (200 μl): (i) 8 mM sodium formate, 0.05 mM ATP, 2 mM MgCl_2_, 100 nM *Pa*FtfL, and different amounts of THF (32.5, 65, 130, 325, 650, and 1300 μM); (ii) 8 mM sodium formate, 0.04 mM THF, 2 mM MgCl_2_, 100 nM *Pa*FtfL, and ATP (10, 20, 50, 100, and 200 μM). The reaction mixture was incubated at 30 °C for 2 min. Then, the reaction was terminated by adding HCl (0.36 M, 2 × volume of the reaction mixture). The inhibition constants (*K*_i_) of BBR on *Pa*FtfL based on THF or ATP were determined by measuring the apparent *K*_m_ with the addition of 40 μM BBR into the reaction mixture.

### Crystallization and structure determination

The crystals of *Pa*FtfL apo were obtained by using a sitting drop vapor diffusion method at 22 °C with 1 μl drops containing a 1:1 mixture of crystallization buffer (15% w/v PEG 3350, 0.15 M cesium chloride) and 10 mg/ml protein. The crystals were grown for 7 days and then applied for X-ray diffraction data collection. Before frozen in liquid nitrogen, crystals were stabilized in cryoprotectant (0.15 M cesium chloride, 17.5% v/v ethylene glycol, and 15% w/v PEG 3350).

*Pa*FtfL-ATP crystals were obtained by soaking *Pa*FtfL apo crystals in crystallization buffer (200 mM potassium formate, 20% w/v PEG 3350) containing 1 mM ATP. Before frozen in liquid nitrogen, crystals were stabilized in cryoprotectant (200 mM potassium formate, 20% w/v PEG 3350, and 12.5% v/v 1,2-butanediol).

For *Pa*FtfL-BBR crystals preparation, the *Pa*FtfL protein in 10 mg/ml was mixed with 1 mM BBR (stock in 100 mM, 100% DMSO) and then incubated at 25 °C for 1 h. The crystals were obtained by a sitting drop vapor diffusion method at 22 °C with 1 μl drops containing a 1:1 mixture of 10 mg/ml *Pa*FtfL-BBR mixture and crystallization buffer (2.0 M ammonium sulfate, 0.1 M HEPES pH 7.5), The crystals were grown for 7 days before X-ray diffraction data collection. Before frozen in liquid nitrogen, crystals were stabilized in cryoprotectant (2.0 M ammonium sulfate, 0.1 M HEPES pH 7.5, and 3 M l-proline). Data were collected at Shanghai Synchrotron Radiation Facility (SSRF) beamlines 02U1 and then were processed using XDS [40] for *Pa*FtfL apo and *Pa*FtfL-BBR datasets and xia2-3dii for *Pa*FtfL-ATP dataset. The molecular replacement was performed by MorDa [41] at CCP4 online server (https://ccp4online.ccp4.ac.uk/). The model building and refinement were performed in Coot [42] and Phenix [43].

### Phylogenetic analysis of FtfL homologs

The amino acid sequences of FtfL homologs were obtained by using the TBLASTN search. The obtained protein was aligned with Clustal W software. The alignment was visualized with MEGA 7 program [44]. The maximum likelihood phylogenetic tree was generated from this alignment in MEGA7. Finally, annotation was made manually.

### Determination of FtfL abundance using healthy human metagenomic data

All the amino acid sequences from homologous enzymes in the phylogenetic tree were used to generate the SSNs [45], using the EFI-EST webtool (http://efi.igb.illinois.edu/efi-est/). Then, the network was generated with initial edge values of 215 as previously reported [46]. The abundance of FtfLs in human metagenomes was obtained by using ShortBRED.

### Supplementary Information

**Additional file 1: Fig. S1.** The effect of BBR on the growth of probiotics. **Fig. S2.** Synthesis and validation of the probe. **Fig. S3.** The pull-down experiments between BBR and candidate proteins from *P. anaerobius*. **Fig. S4.** BBR doesn’t bind *Pa*EF4 by BLI. **Fig. S5.** The analysis of crystal structures of *Pa*FtfL apo enzyme, *Pa*FtfL-ATP, and *Pa*FtfL-BBR. **Fig. S6.** The enzyme kinetics measurement of *Pa*FtfL. **Fig. S7.** Residual activity of *Pa*FtfL derivatives compared to wild-type *Pa*FtfL. **Fig. S8.** The IC_50_ values of BBR inhibition on different FtfL enzymes in this study. **Fig. S9.** The expression of *hs*MTHFD1L and *hs*MTHFD1 across human body. **Fig. S10.** Alignment of *Pa*FtfL, *hs*MTHFD1 and *hs*MTHFD1L. **Table S1.** Primers used in this work. **Table S2.** Plasmids used in this work. **Table S3.** Top proteins significantly enriched by BBP. **Table S4.** Data collection and model refinement statistics. **Table S5.** Enzyme kinetic parameters for *Pa*FtfL enzymes. **Table S6.** The Bio-Layer Interferometer assay showing binding affinities of BBR to derivatives of *Pa*FtfL.

## Data Availability

All data are available in the main text or the supplementary materials. All the primers and plasmids used in this study were listed in Tables S1 and S2 respectively. The atomic coordinates of FtfL-apo, FtfL-ATP, and FtfL-BBR structures have been deposited in the Protein Data Bank with accession codes of 7XZN [47], 7XZO [48], and 7XZP [49], respectively. The crystal structure of *Mt*FtfL-folate is available in PDB, accession number: 4JJK [50]. The protein sequences of predicted or characterized FtfL were downloaded from the NCBI database (https://www.ncbi.nlm.nih.gov/). The data on human gutmetagenomes were obtained from the ShortBRED database (https://huttenhower.sph.harvard.edu/shortbred/).

## Abbreviations

BBR: Berberine
CRC: Colorectal cancer
FtfL: Formate tetrahydrofolate ligase
mTOR: Mammalian target of rapamycin
RXRα: Retinoid X receptor α
AMPK: Adenosine monophosphate-activated protein kinase
SCFA: Short-chain fatty acids
BBP: BBR-biotinylated probe
LC-MS: Liquid chromatography-tandem mass spectrometry
EF4: Elongation factor 4
BLI: Bio-layer interferometry
*Kd*: Dissociation constant
IC_50_: The half maximal inhibitory concentration
*K*_m_: Michaelis constant
SSN: Sequence similarity network
MTHFD1: Methylenetetrahydrofolate dehydrogenase 1
MTHFD1L: Methylenetetrahydrofolate dehydrogenase 1-like
G4s: G quadruplexes
UHRF1: Ubiquitin-like with PHD and RING finger domains
MAP2K7: Mitogen-activated protein kinase 7
FtsZ: Filamentation temperature sensitive protein Z
*K*_i_: Inhibition constants
