# Supplementary material for: Identification of FtfL as a novel target of berberine in intestinal bacteria
Source: BMC Biol. 2023 Dec 5;21:280. doi: 10.1186/s12915-023-01778-w (PMC10696740; doi:10.1186/s12915-023-01778-w)
Supplement: Supplementary file 1 — Additional file 1: Fig. S1. The effect of BBR on the growth of probiotics. Fig. S2. Synthesis and validation of the probe. Fig. S3. The pull-down experiments between BBR and candidate proteins from P. anaerobius. Fig. S4. BBR doesn’t bind PaEF4 by BLI. Fig. S5. The analysis of crystal structures of PaFtfL apo enzyme, PaFtfL-ATP, and PaFtfL-BBR. Fig. S6. The enzyme kinetics measurement of PaFtfL. Fig. S7. Residual activity of PaFtfL derivatives compared to wild-type PaFtfL. Fig. S8. The IC50 values of BBR inhibition on different FtfL enzymes in this study. Fig. S9. The expression of hsMTHFD1L and hsMTHFD1 across human body. Fig. S10. Alignment of PaFtfL, hsMTHFD1 and hsMTHFD1L. Table S1. Primers used in this work. Table S2. Plasmids used in this work. Table S3. Top proteins significantly enriched by BBP. Table S4. Data collection and model refinement statistics. Table S5. Enzyme kinetic parameters for PaFtfL enzymes. Table S6. The Bio-Layer Interferometer assay showing binding affinities of BBR to derivatives of PaFtfL. [file 12915_2023_1778_MOESM1_ESM.docx]

**Supplementary Figures**

**Figure S1**


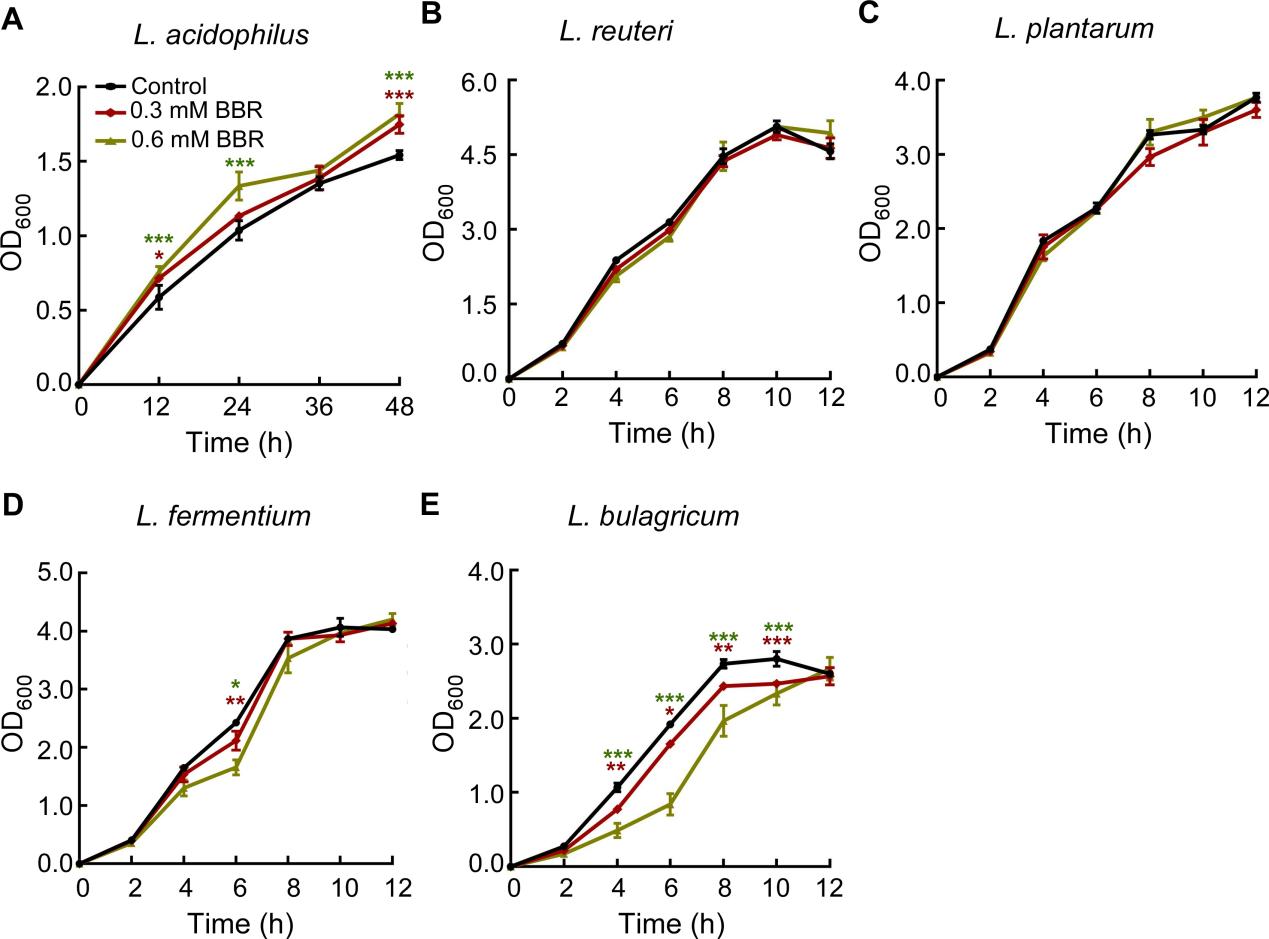


**Figure S1. The effect of BBR on the growth of probiotics. A-E**, BBR promotes growth of *L. acidophilus* ATCC 4356, inhibits growth of *L. bulagricum* BD 15, and has little effect on *L. reuteri* DSM 17938, *L. plantarum* DSM 13171 and *L. fermentium* CGMCC 1.1880. Data are presented as the mean ± S.D. of three independent replicates. The statistical significance was assessed by the two-way ANOVA with Tukey’s multiple comparison test. (****p*<0.001; ***p*<0.01; **p*<0.05).

**Figure S2**


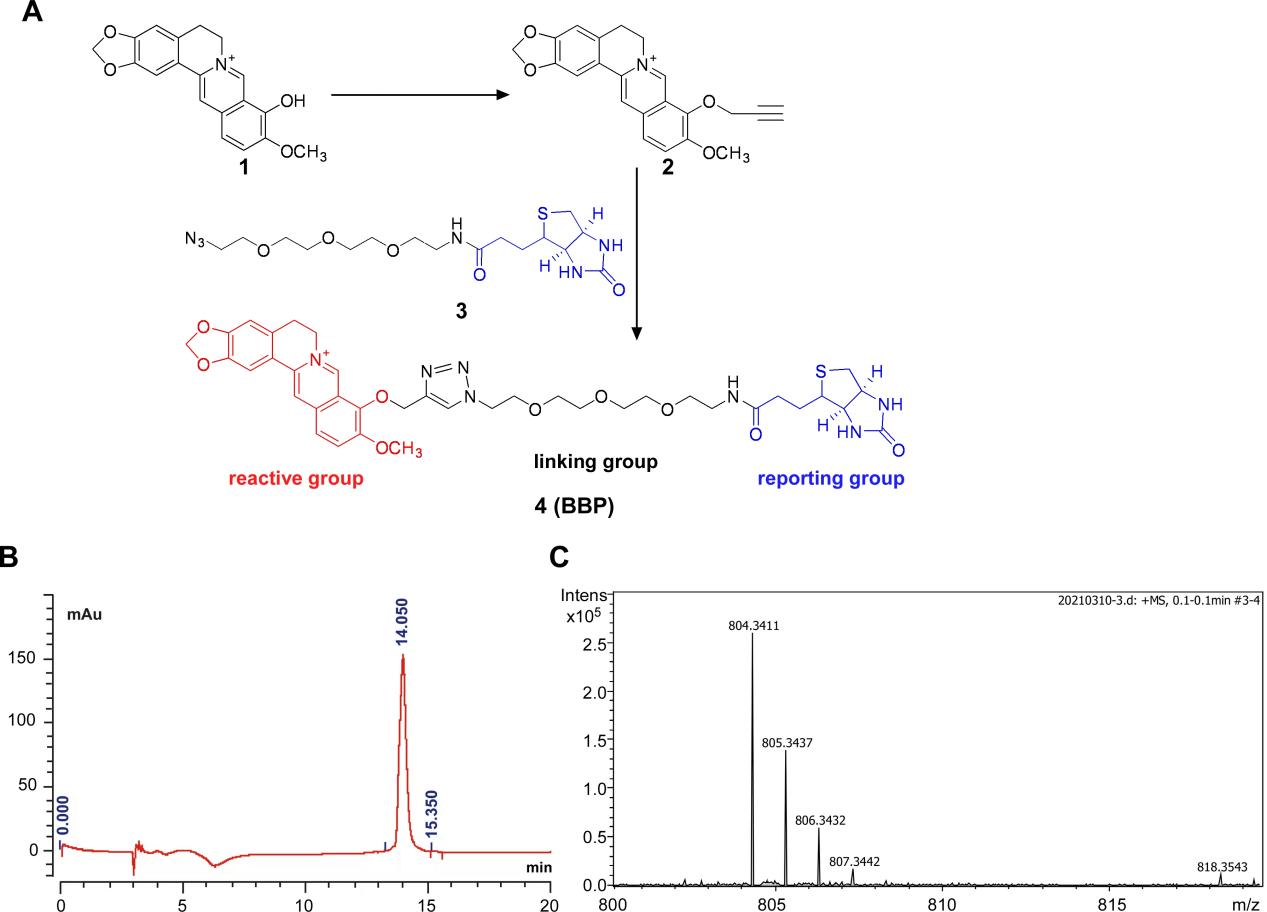


**Figure S2. Synthesis and validation of the probe. A,** Synthesis of the chemical probe, BBP. **B-C,** Identification of the probe by LC-MS. The purity of the BBP (C_40_H_50_N_7_O_9_S^+^) was identified by LC-MS. The retention time of BBP is 14.05 min, and the molecular weight is 804.3411.

**Figure S3**


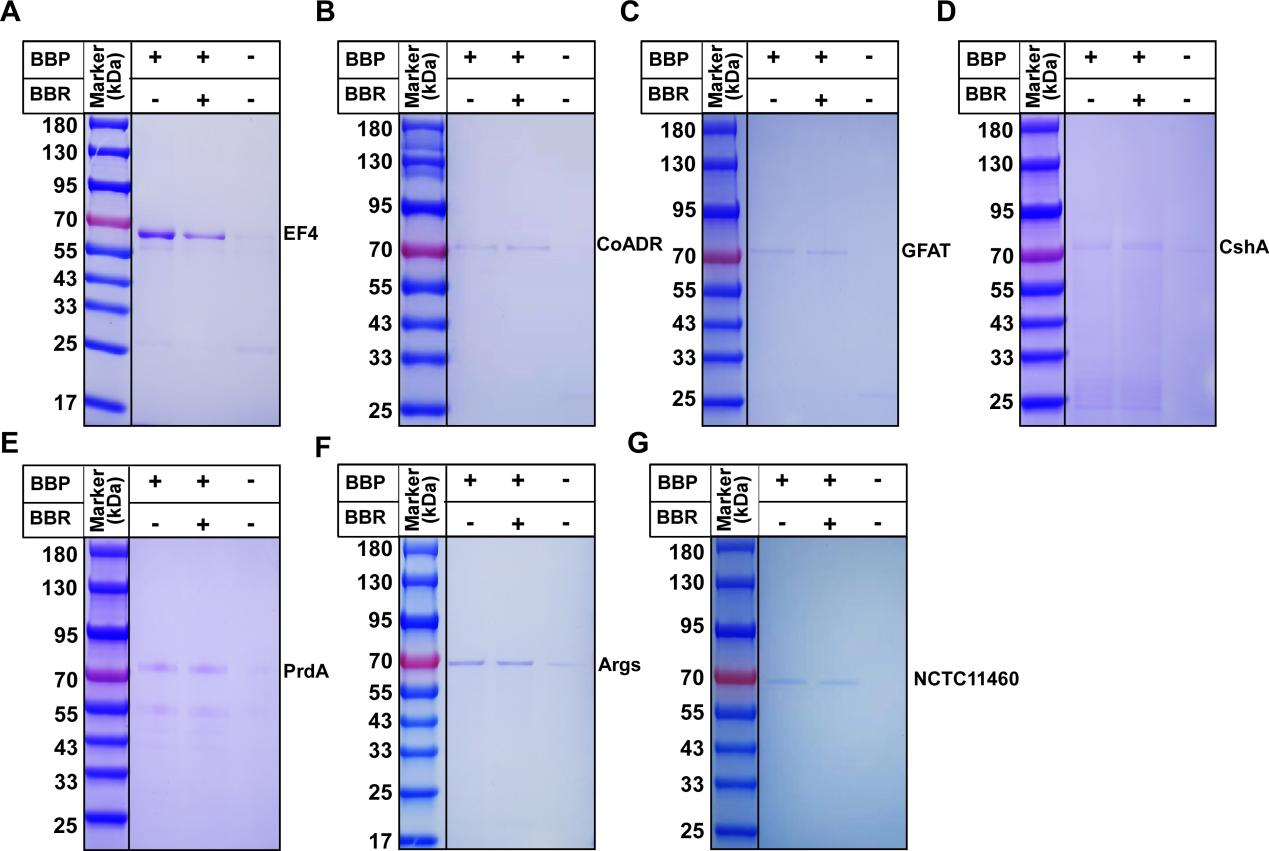


**Figure S3. The pull-down experiments between BBR and candidate proteins from *P. anaerobius*.** BBP (100 μM) was incubated with candidate proteins (3 μM) with or without BBR (1 mM). After washing and centrifugating to remove nonbound components, eluted proteins were separated by SDS-PAGE.

**Figure S4**

**
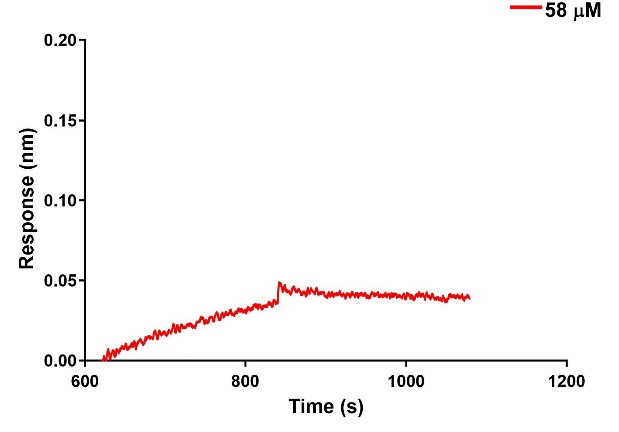
**

**Figure S4. BBR doesn’t bind *Pa*EF4 by BLI.** The interaction of BBR to *Pa*EF4 was measured by BLI.

**Figure S5**


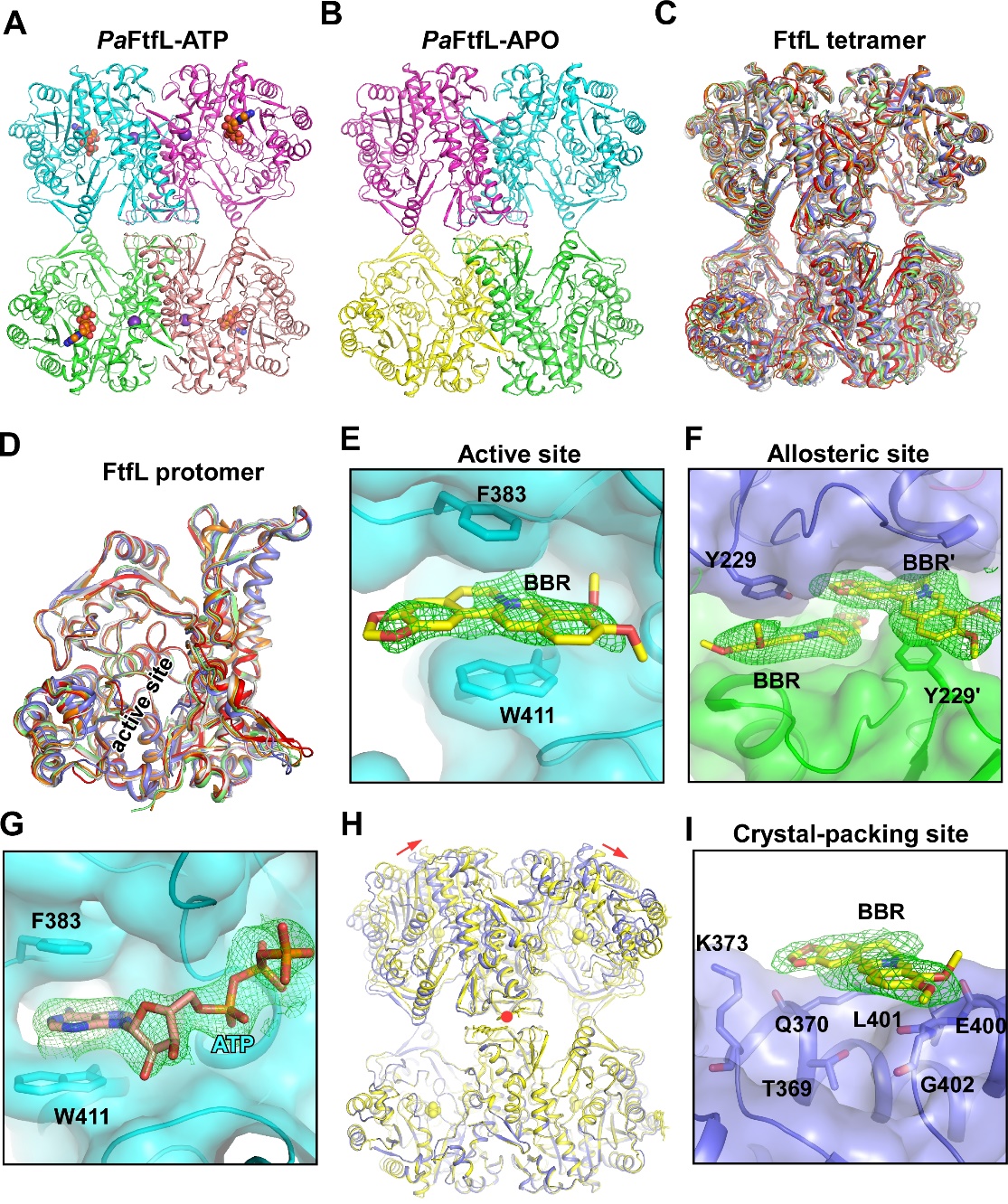


**Figure S5.** **The analysis of crystal structures of *Pa*FtfL apo enzyme, *Pa*FtfL-ATP, and *Pa*FtfL-BBR. A,** The overall structure of *Pa*FtfL-ATP. The four protomers are in different colors. **B,** The overall structure of *Pa*FtfL apo enzyme. **C,** Structure superimposition of *Pa*FtfL apoenzyme (red) with *M. extorquens* FtfL (gray, PDB: 7C11), *T. maritima* FtfL (blue, PDB: 3DO6), *M. thermoacetica* FtfL (orange, PDB: 4JIM), *T. acetatoxydans* Re1 FtfL (green, PDB: 5A4J). **D,** Structural comparison reveals that *Pa*FtfL adopts essentially the same overall fold of the protomers and exhibits identical active sites as reported crystal structures of FtfL. The colors are as above. **E,** The interaction of BBR with FtfL in its active site, green mesh, the *Fo-Fc* electron density difference map of BBR contoured at 2.0 σ.**F,** The interaction of BBR with FtfL in its allosteric site.  **G,** The interaction of ATP with FtfL in its active site. Green mesh, the Fo-Fc electron density difference map contoured at 2.0 σ.**H,** structure superimposition between *Pa*FtfL apoenzyme (blue) and *Pa*FtfL-BBR (yellow) reveals that the presence of BBR in the interface causes a 4.4º rotation of one dimer towards the other. I, The interaction of BBR with FtfL in the crystal-packing site.

**Figure S6**


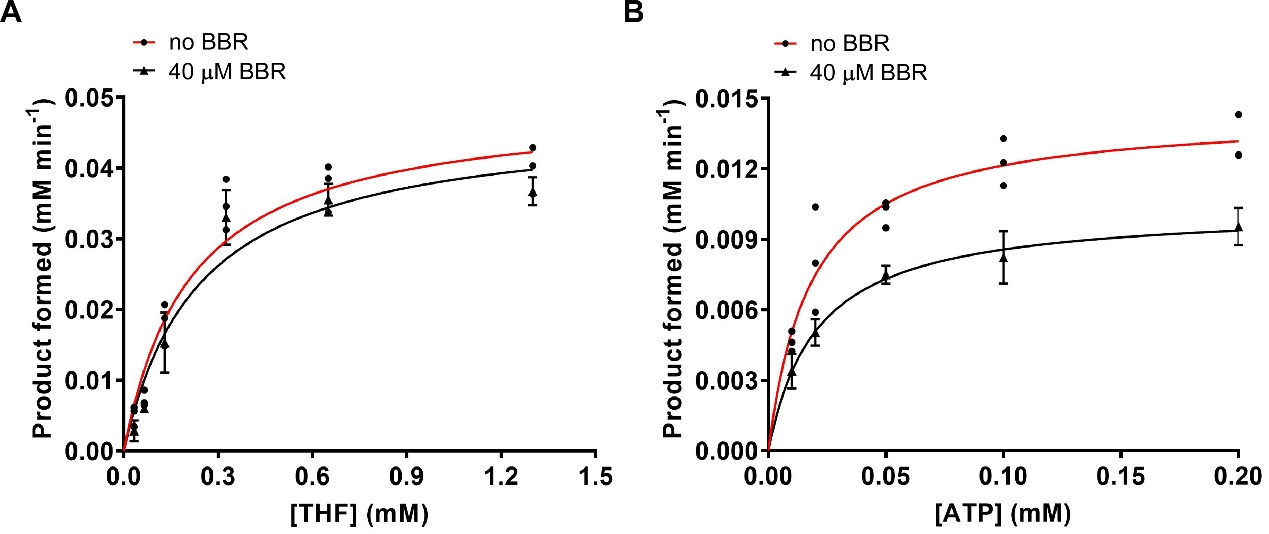


**Figure S6.** **The enzyme kinetics measurement of *Pa*FtfL. A**, The determination of THF *K*_m_, *K*_cat_, *V*_max_ for *Pa*FtfL and the determination of THF apparent *K*_m_, *K*_cat_, *V*_max_ for *Pa*FtfL in the presence of 40 μM BBR. **B**, The determination of ATP *K*_m_, *K*_cat_, *V*_max_ for *Pa*FtfL and the determination of ATP apparent *K*_m_, *K*_cat_, *V*_max_ for *Pa*FtfL in the presence of 40 μM BBR. Data are presented as means ± S.D. of 3 independent replicates. Enzyme-specific activity values were calculated based on the *V*_max_ values obtained via Michaelis-Menten or Hill fitting. The one-way ANOVA test was used for statistical analysis, *p*<0.01.

**Figure S7**

**
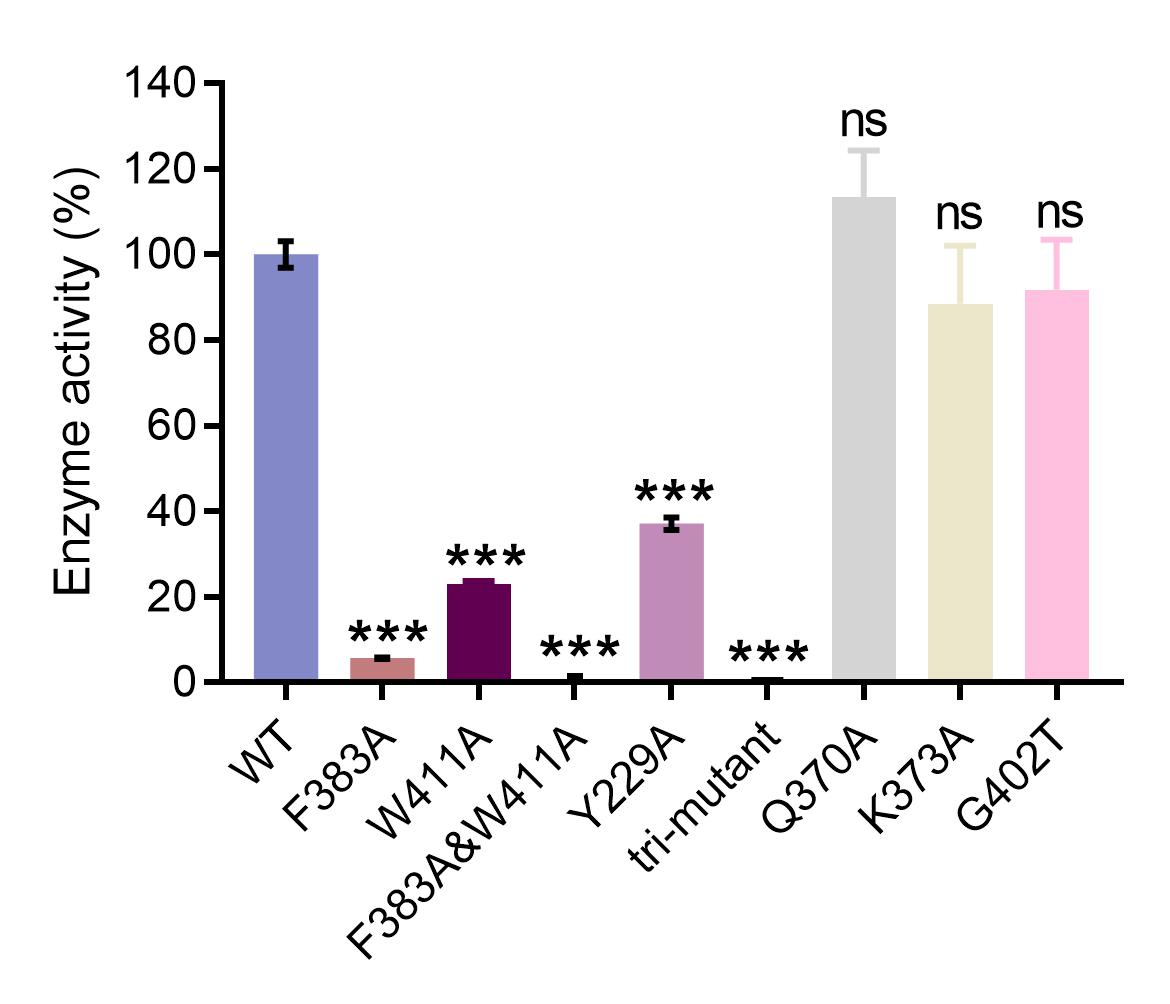
**

**Figure S7. Residual activity of *Pa*FtfL derivatives compared to wild-type *Pa*FtfL.** Tri-mutant represents a three-amino acid mutant of *Pa*FtfL (Y229A&F383A&W411A). Each bar represents the mean ± S.D. of the data from two independent experiments. The statistical significance of the phenotypic differences between the activities of the WT and derivative enzymes were assessed by the one-way ANOVA with Tukey’s multiple comparison test. (****p*<0.001; ***p*<0.01; **p*<0.05).

**Figure S8**

**
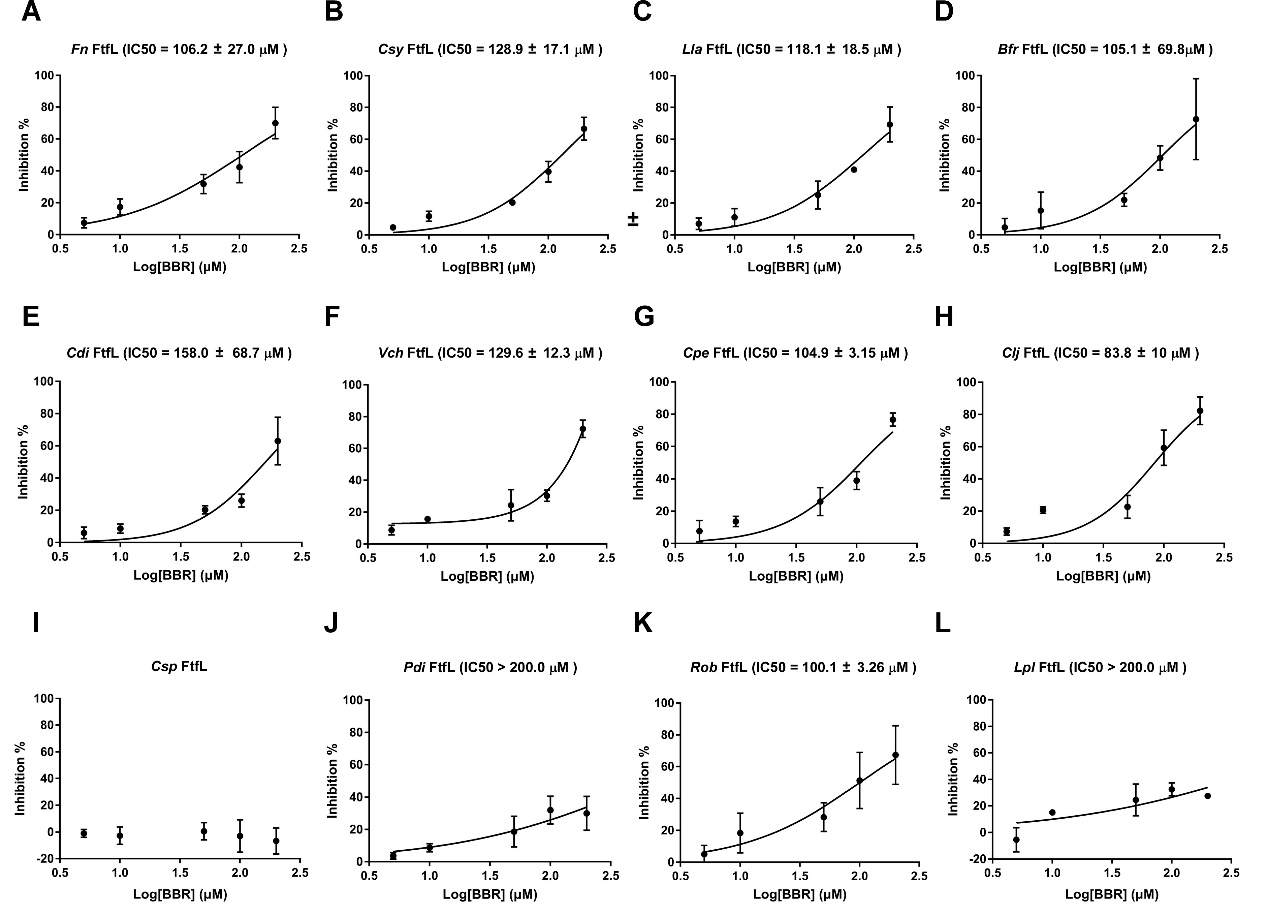
**

**Figure S8. The IC_50_ values of BBR inhibition on different FtfL enzymes in this study.** The dose-dependent inhibitory curves were fit to calculate the IC_50_ values. Data shown are mean ± S.D. from three independent experiments. The one-way ANOVA test was used for statistical analysis, *p*<0.01.

**Figure S9**


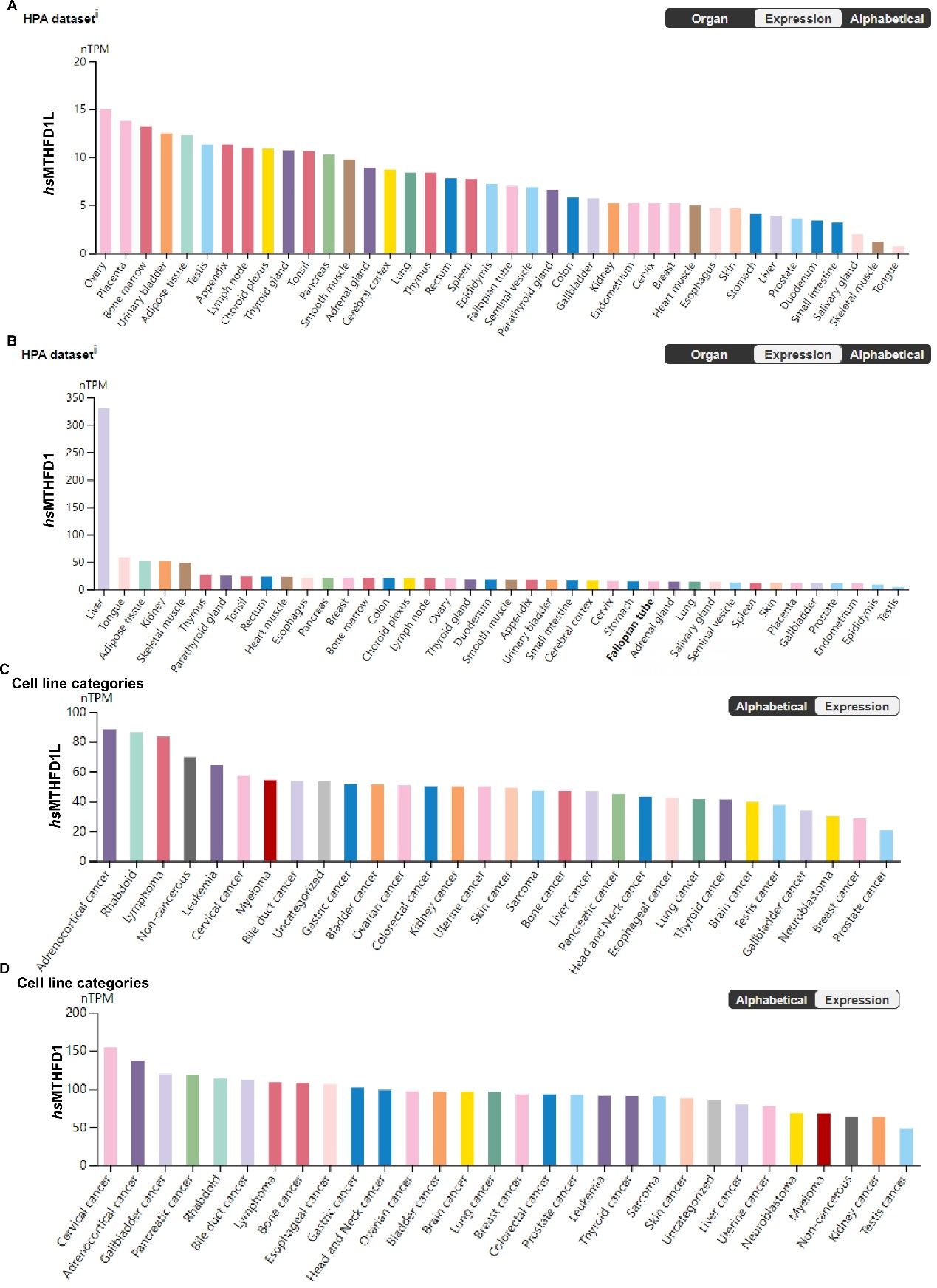


**Figure S9. The expression of *hs*MTHFD1L and *hs*MTHFD1 across human body.** **A, B** The expression levels of *hs*MTHFD1L (A) and *hs*MTHFD1 (B) in various normal tissues. **C, D** The expression levels of *hs*MTHFD1L (C) and *hs*MTHFD1 (D) in various cancer tissues.

**Figure S10**


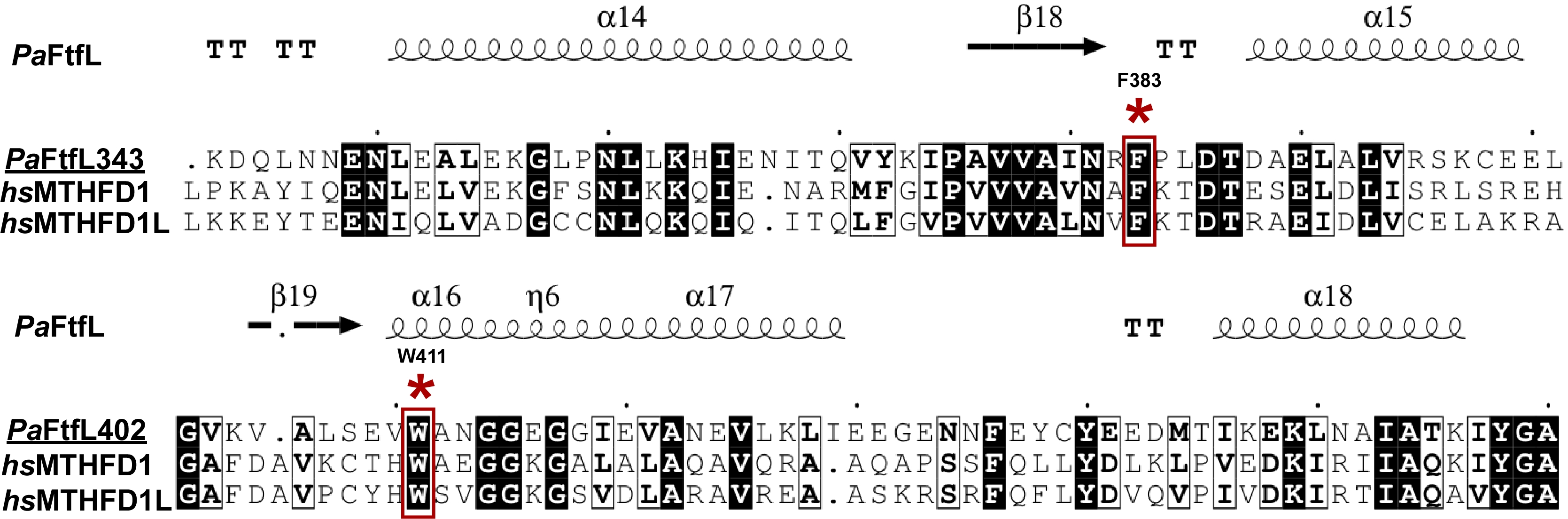


**Figure S10.** **Alignment of *Pa*FtfL, *hs*MTHFD1 and *hs*MTHFD1L.** The residues play a role in BBR binding are labeled by red asterisk. The numbering of residues is according to *Pa*FtfL. Since *hs*MTHFD1 and *hs*MTHFD1L could be homodimers (PDB: 1DIJ), the differences on the allosteric site were not considered.

**Supplementary Tables**

**Table S1. Primers used in this work.**

| **Names** | **Sequences (5'-3')** |
| --- | --- |
| 27-F | AGAGTTTGATCCTGGCTCAG |
| 1492-R | GGTTACCTTGTTACGACTT |
| *Pa*ftfl-F | CGGCCTGGTGCCGCGCGGCAGCCATATGATGGAATTTAAGACAGATATTGAAATAGCA |
| *Pa*ftfl -R | GGATCTCAGTGGTGGTGGTGGTGGTGCTCGAGCTAGAATAGACCTGATATCTTTCCATT |
| *Pa*cdr-F | AGCGGCCTGGTGCCGCGCGGCAGCCATATGATGAAAAAGATATTAATAGTTGGTGG |
| *Pa*cdr-R | TCTCAGTGGTGGTGGTGGTGGTGCTCGAGCTATATTATGTTCTCATCATTAATTACTTT |
| *Pa*lepA-F | CAGCGGCCTGGTGCCGCGCGGCAGCCATATGTTGACAAATAGACAAAGTAGAACTAG |
| *Pa*lepA-R | GGATCTCAGTGGTGGTGGTGGTGGTGCTCGAGTTACTCATCCAATTTAAGAACAGACAT |
| *Pa*NCTC11460-F | AGCAGCGGCCTGGTGCCGCGCGGCAGCCATATGATGAAAAAAGTACGTTATGAAGAACC |
| *Pa*NCTC11460-R | CGGATCTCAGTGGTGGTGGTGGTGGTGCTCGAGTTACTCGTTCAAGCTAAGTATTGACG |
| *Pa*arg-F | AGCAGCGGCCTGGTGCCGCGCGGCAGCCATATGATGGCAGATTTCAAGCAAGTGAT |
| *Pa*arg-R | CGGATCTCAGTGGTGGTGGTGGTGGTGCTCGAGCTACATCTTCTCAGGTGCTTTCATAC |
| *Pa*glms-F | AGCGGCCTGGTGCCGCGCGGCAGCCATATGATGTGTGGAATAGTAGGATATTTAGGT |
| *Pa*glms-R | CCGGATCTCAGTGGTGGTGGTGGTGGTGCTCGAGCTATTCAACTGTAACTGATTTAGCA |
| *Pa*deaD-F | AGCGGCCTGGTGCCGCGCGGCAGCCATATGATGGAAATAACAAATTTTAACGAATTG |
| *Pa*deaD-R | GCCGGATCTCAGTGGTGGTGGTGGTGGTGCTCGAGCTACTTGCTAAAACGTCTTCTCTT |
| *Pa*prdA-F | GGCCTGGTGCCGCGCGGCAGCCATATGGTGTCAATTACAAAGGAAACTGTACAGGCTC |
| *Pa*prdA-R | TCTCAGTGGTGGTGGTGGTGGTGCTCGAGTTAGTGAAGTGTTCCACTGTTTACTAGAAC |
| *Fn*ftfl-F | CAGCGGCCTGGTGCCGCGCGGCAGCCATATGATGACTGATATTCAAATTGCACAAGCA |
| *Fn*ftfl -R | CTCAGTGGTGGTGGTGGTGGTGCTCGAGTTAGAATAATCCAGAGATAACCCCATTTTCA |
| *Csy*ftfl -F | GCGGCCTGGTGCCGCGCGGCAGCCATATGATGAAAACAGATATCGAAATTGCACAG |
| *Csy*ftfl -R | CTCAGTGGTGGTGGTGGTGGTGCTCGAGTTAGAACAGACCGGAAATCTTTCCTGTCGC |
| *Lla*ftfl –F | GCGGCCTGGTGCCGCGCGGCAGCCATATGATGAAAACGGATATTGAAATTGCTCAAGCT |
| *Lla*ftfl-R | TCTCAGTGGTGGTGGTGGTGGTGCTCGAGCTAAAATAATCCTGAGATTTTTCCATTAT |
| *Sfa*ftfl –F | CGGCCTGGTGCCGCGCGGCAGCCATATGATGAAGACAGATATTAACATTGCACAAGCA |
| *Sfa*ftfl –R | TCTCAGTGGTGGTGGTGGTGGTGCTCGAGTTAAAAGAGACCGCTCGCTTGACCCGTTTC |
| *Bfr*ftfl-F | GCGGCCTGGTGCCGCGCGGCAGCCATATGATGAAATCAGACATAGAAATAGCACGTAGT |
| *Bfr*ftfl-R | CTCAGTGGTGGTGGTGGTGGTGCTCGAGTTAGCTCAATCCTTCAATATTGCCATCCACA |
| *Cdi*ftfl-F | GCGGCCTGGTGCCGCGCGGCAGCCATATGATGGGATTTAAATCTGACATTGAAATAGCT |
| *Cdi*ftfl-R | TCTCAGTGGTGGTGGTGGTGGTGCTCGAGTTAGAATAATCCAGCTATTACTCCATTTTC |
| *Vch*ftfl-F | AGCGGCCTGGTGCCGCGCGGCAGCCATATGATGAGCGATCTTGAGATTGCCCGCGCCGC |
| *Vch*ftfl-R | ATCTCAGTGGTGGTGGTGGTGGTGCTCGAGCTAGAACAGCCCGACGGCGTTACCCTCGG |
| *Cpe*ftfl-F | CGGCCTGGTGCCGCGCGGCAGCCATATGATGAAAAATGATATTGAAATAGCACAAAGTG |
| *Cpe*ftfl-R | TCTCAGTGGTGGTGGTGGTGGTGCTCGAGTTAGAATAATCCTACTATTTCTCCATTGTC |
| *Clj*ftfl-F | GGCCTGGTGCCGCGCGGCAGCCATATGATGACTTATAAATCAGACATCGAAATAGCTCA |
| *Clj*ftfl-R | TCTCAGTGGTGGTGGTGGTGGTGCTCGAGTTAGAATAATCCGCTTATTACTCCATTTTC |
| *Csp*ftfl-F | CGGCCTGGTGCCGCGCGGCAGCCATATGATGTTTAAATCCGATATAGAAATAGCACAAG |
| *Csp*ftfl-R | TCAGTGGTGGTGGTGGTGGTGCTCGAGTTAGAATAGTCCAACTATATTGCCATCTGATA |
| *Pdi*ftfl-F | GCGGCCTGGTGCCGCGCGGCAGCCATATGATGAAATCGGACATTGAGATAGCAAGAGAA |
| *Pdi*ftfl-R | TCTCAGTGGTGGTGGTGGTGGTGCTCGAGTTAACTCAATCCCTCAATCATGCCATCCAC |
| *Rob*ftfl-F | GCGGCCTGGTGCCGCGCGGCAGCCATATGATGGGATTCAAAAGCGACATCGAAATTGCA |
| *Rob*ftfl-R | CTCAGTGGTGGTGGTGGTGGTGCTCGAGTTAGAAAAGTCCGGTAATCTTTCCTGTCTCA |
| *Lac*ftfl –F | AGCAGCGGCCTGGTGCCGCGCGGCAGCCATATGATGCCAACTGACATTGAAATTGCTGA |
| *Lac*ftfl –R | GATCTCAGTGGTGGTGGTGGTGGTGCTCGAGTTAAAATAATCCTGCAATTTGACCATT |
| *Lre*ftfl –F | GCGGCCTGGTGCCGCGCGGCAGCCATATGATGACAGACATTGAGATTGCAGACCAAGCA |
| *Lre*ftfl –R | TCTCAGTGGTGGTGGTGGTGGTGCTCGAGTTAGAATAATCCGGTAATTTTACCATTATC |
| *Lpl*ftfl –F | AGCGGCCTGGTGCCGCGCGGCAGCCATATGATGAAGGATATTGAAATTGCACAGCAAGT |
| *Lpl*ftfl –R | TCTCAGTGGTGGTGGTGGTGGTGCTCGAGTTAGAACAAACCTGAGATTTTTCCATCATC |
| *Lfe*ftfl –F | GCGGCCTGGTGCCGCGCGGCAGCCATATGGTGTTAACTGATATTGAAATTGCTGATCAA |
| *Lfe*ftfl –R | CTCAGTGGTGGTGGTGGTGGTGCTCGAGCTAAAATAACCCCGTTATCTTCCCCTCGGAA |
| *Hs*mthfd1-F | CGGCCTGGTGCCGCGCGGCAGCCATATGATGGCGCCAGCAGAAATCCTGAACGGGAAGG |
| *Hs*mthfd1-R | CTCAGTGGTGGTGGTGGTGGTGCTCGAGTTATACATGATGAGAATTAAGGCTGAAAATG |
| *Hs*mthfd1L-F | GCGGCCTGGTGCCGCGCGGCAGCCATATGATGCAATCGCGCCGGGCGCGACCCAGACGA |
| *Hs*mthfd1L-R | TCTCAGTGGTGGTGGTGGTGGTGCTCGAGTTAGAACAAGCCTTTAACTTGTTCTGTTC |
| *Pa*ftfl(Y229A)-F | GTAGTTGCCTACAATGCTTCAGGTAAGCCTGTA |
| *Pa*ftfl(Y229A)-R | GTAGTTGCCTACAATGCTTCAGGTAAGCCTGTA |
| *Pa*ftfl(F383A)-F | GTTGCAATCAATAGAGCCCCACTAGATACTGAT |
| *Pa*ftfl(F383A)-R | GGCTCTATTGATTGCAACTACTGCTGGTATCTT |
| *Pa*ftfl(W411A)-F | GCCCTATCAGAAGTTGCGGCAAATGGTGGAGA |
| *Pa*ftfl(W411A)-R | CGCAACTTCTGATAGGGCAACCTTTACACCTAA |
| *Pa*ftfl(Q370A)-F | ATAGAGAATATAACTGCGGTATACAAGATACCA |
| *Pa*ftfl(Q370A)-R | CGCAGTTATATTCTCTATATGCTTTAACAG |
| *Pa*ftfl(K373A)-F | ATAACTCAGGTATACGCGATACCAGCAGTAGTT |
| *Pa*ftfl(K373A)-R | CGCGTATACCTGAGTTATATTCTCTATATG |
| *Pa*ftfl(G402T)-F | AAGTGTGAAGAATTAACTGTAAAGGTTGCCCTA |
| *Pa*ftfl(G402T)-R | AGTTAATTCTTCACACTTAGACCTAACAAG |

**Table S2. Plasmids used in this work.**

| **Plasmids** | **Genotypes** | **Sources** |
| --- | --- | --- |
| pET28a | Km^r^; T7 promoter | Novagen |
| pET28a-*Pa*FtfL | Vector for expressing the *Pa*ftfL gene; Km^r^; T7 promoter | This study |
| pET28a-*Pa*CoADR | Vector for expressing the *Pa*cdr gene; Km^r^; T7 promoter | This study |
| pET28a-*Pa*EF4 | Vector for expressing the *Pa*lepA gene; Km^r^; T7 promoter | This study |
| pET28a-*Pa*NCTC | Vector for expressing the *Pa*NCTC11460 gene; Km^r^; T7 promoter | This study |
| pET28a-*Pa*Args | Vector for expressing the *Pa*args gene; Km^r^; T7 promoter | This study |
| pET28a-*Pa*GFAT | Vector for expressing the *Pa*glms gene; Km^r^; T7 promoter | This study |
| pET28a-*Pa*CshA | Vector for expressing the *Pa*deaD gene; Km^r^; T7 promoter | This study |
| pET28a-*Pa*PrdA | Vector for expressing the *Pa*prdA gene; Km^r^; T7 promoter | This study |
| pET28a-*Fn*FtfL | Vector for expressing the *Fn*ftfL gene; Km^r^; T7 promoter | This study |
| pET28a-*Csy*FtfL | Vector for expressing the *Csy*ftfL gene; Km^r^; T7 promoter | This study |
| pET28a-*Lla*FtfL | Vector for expressing the *Lla*ftfL gene; Km^r^; T7 promoter | This study |
| pET28a-*Sfa*FtfL | Vector for expressing the *Sfa*ftfL gene; Km^r^; T7 promoter | This study |
| pET28a-*Bfr*FtfL | Vector for expressing the *Bfr*ftfL gene; Km^r^; T7 promoter | This study |
| pET28a-*Cdi*FtfL | Vector for expressing the *Cdi*ftfL gene; Km^r^; T7 promoter | This study |
| pET28a-*Vch*FtfL | Vector for expressing the *Vch*ftfL gene; Km^r^; T7 promoter | This study |
| pET28a-*Cpe*FtfL | Vector for expressing the *Cpe*ftfL gene; Km^r^; T7 promoter | This study |
| pET28a-*Clj*FtfL | Vector for expressing the *Clj*ftfL gene; Km^r^; T7 promoter | This study |
| pET28a-*Csp*FtfL | Vector for expressing the *Csp*ftfL gene; Km^r^; T7 promoter | This study |
| pET28a-*Pdi*FtfL | Vector for expressing the *Pdi*ftfL gene; Km^r^; T7 promoter | This study |
| pET28a-*Rob*FtfL | Vector for expressing the *Rob*ftfL gene; Km^r^; T7 promoter | This study |
| pET28a-*Lac*FtfL | Vector for expressing the *Lac*ftfL gene; Km^r^; T7 promoter | This study |
| pET28a-*Lre*FtfL | Vector for expressing the *Lre*ftfL gene; Km^r^; T7 promoter | This study |
| pET28a-*Lpl*FtfL | Vector for expressing the *Lap*ftfL gene; Km^r^; T7 promoter | This study |
| pET28a-*Lfe*FtfL | Vector for expressing the *Lfe*ftfL gene; Km^r^; T7 promoter | This study |
| pET28a-*hs*MTHFD1 | Vector for expressing the *hs*mthfd1 gene; Km^r^; T7 promoter | This study |
| pET28a-*hs*MTHFD1L | Vector for expressing the *hs*mthfd1L gene; Km^r^; T7 promoter | This study |
| pET28a-*Pa*FtfLY229A | Vector for expressing the mutated *PA*ftfL gene (Y229A); Km^r^; T7 promoter | This study |
| pET28a-*Pa*FtfLF383A | Vector for expressing the mutated *Pa*ftfL gene (F383A); Km^r^; T7 promoter | This study |
| pET28a-*Pa*FtfLW411A | Vector for expressing the mutated *Pa*ftfL gene (W411A); Km^r^; T7 promoter | This study |
| pET28a-*Pa*FtfLQ370A | Vector for expressing the mutated *Pa*ftfL gene (Q370A); Km^r^; T7 promoter | This study |
| pET28a-*Pa*FtfLK373A | Vector for expressing the mutated *Pa*ftfL gene (K373A); Km^r^; T7 promoter | This study |
| pET28a-*Pa*FtfLG402T | Vector for expressing the mutated *Pa*ftfL gene (G402T); Km^r^; T7 promoter | This study |

**Table S3. Top proteins significantly enriched by BBP.**

| **ID** | **Gene** | **Protein** | **Ratio iBAQ(BBP/BBR)** |
| --- | --- | --- | --- |
| R5J165 | *ftfl* | Formate--tetrahydrofolate ligase | 10.07 |
| R5J852 | *cdr* | Coenzyme A disulfide reductase | 5.55 |
| R5JGR7 | *lepA* | Elongation factor 4 | 4.85 |
| R5J8G7 | *NCTC11460* | Uncharacterized protein | 4.25 |
| R5J0G2 | *args* | Arginine--tRNA ligase | 3.83 |
| R5J5B9 | *glms* | Glutamine--fructose-6-phosphate  aminotransferase | 3.58 |
| R5J5B9 | *deaD* | Cold-shock DEAD box protein A | 2.83 |
| A0A379CIZ8 | *prdA* | D-proline reductase proprotein prdA | 2.28 |

*Ratio iBAQ (BBP/BBR) represents BBR competitive effect were selected based on the intensity ratio of the hits between the experimental group and competitive group.

**Table S4. Data collection and model refinement statistics.**

|  | ***Pa*FtfL apo** | ***Pa*FtfL-ATP** | ***Pa*FtfL-BBR** |
| --- | --- | --- | --- |
| **Data collection** |  |  |  |
| Space group | *C*1 2 1 | *C*1 2 1 | *C*2 2 2_1_ |
| Cell dimensions |  |  |  |
| a, b, c (Å) | 215.9, 116.3, 107.3 | 215.3, 115.1, 106.5 | 136.26, 205.21, 107.61 |
| α, β, γ (°) | 90.0, 92.6, 90.0 | 90.0, 93.2, 90.0 | 90.0, 90.0, 90.0 |
| Resolution (Å) | 50.00 - 2.04 (2.09-2.04) | 50.00-2.31(2.37-2.31) | 50.00 – 2.56 (2.63-2.56) |
| R_sym_ or R_merge_ | 0.058 (0.720) | 0.119 (0.781) | 0.124 (0.806) |
| I/σI | 8.0 (1.2) | 5.6 (1.2) | 8.8 (1.2) |
| Completeness (%) | 99.9 (100.0) | 99.9 (99.9) | 100.0 (100.0) |
| Redundancy | 8.0 (8.2) | 6.9 (7.2) | 8.0 (8.2) |
| CC_1/2_ in highest shell | 0.998 | 0.995 | 0.978 |
| **Refinement** |  |  |  |
| Resolution (Å) | 30.50 -2.04 | 30.03 – 2.31 | 47.6 - 2.56 |
| No. reflections | 167862 | 113275 | 48808 |
| Rwork/ Rfree | 0.271/0.282 | 0.256/0.290 | 0.201/0.236 |
| No. of atoms |  |  |  |
| B-factors (Å^2^) |  |  |  |
| Macromolecules | 54.49 | 42.33 | 54.61 |
| Ligands | 63.03 | 57.93 | 63.07 |
| R.m.s deviations |  |  |  |
| Bond lengths (Å) | 0.002 | 0.002 | 0.004 |
| Bond angles (°) | 0.573 | 0.499 | 0.767 |
| Ramachandran plot |  |  |  |
| Favored (%) | 96.25 | 96.09 | 97.21 |
| Allowed (%) | 3.7 | 3.73 | 2.7 |
| Disallowed (%) | 0.05 | 0.18 | 0.09 |

**Table S5. Enzyme kinetic parameters for *Pa*FtfL enzymes.**

| *Pa*FtfL | Substrate  (No BBR) | *K*_m_ (mM) | *K*_cat_ (s^-1^) | *V*_max_ (mM/min) |
| --- | --- | --- | --- | --- |
|  | THF | 0.21 ± 0.04 | 81.7 ± 5.0 | 0.049 ± 0.003 |
|  | ATP | 0.018 ± 0.003 | 23.3 ± 5.0 | 0.014 ± 0.003 |
|  | Substrate  (40 μM BBR) | *K*_m_ (mM) | *K*_cat_ (s^-1^) | *V*_max_ (mM/min) |
|  | THF | 0.24 ± 0.06 | 78.3 ± 6.7 | 0.047 ± 0.004 |
|  | ATP | 0.021 ± 0.003 | 16.7 ± 0.83 | 0.010 ± 0.0005 |

**Table S6. The Bio-Layer Interferometer assay showing binding affinities of BBR to derivatives of *Pa*FtfL.**

| *Pa*FtfL or *Pa*FtfL derivatives | Binding affinity of BBR with *Pa*FtfL or *Pa*FtfL derivatives |
| --- | --- |
| Wildtype | *Kd* = 674.0 ± 46.4 nM |
| F383A | *Kd* = 23.1 ± 1.7 μM |
| W411A | *Kd* = 3.8 ± 0.2 μM |
| Y229A | The affinity is too weak to fit |
| Y229A/F383A/W411A | non-detectable |
